# Mesoscale Brain Mapping: Bridging Scales and Modalities in Neuroimaging – A Symposium Review

**DOI:** 10.1007/s12021-024-09686-2

**Published:** 2024-09-23

**Authors:** Joshua K. Marchant, Natalie G. Ferris, Diana Grass, Magdelena S. Allen, Vivek Gopalakrishnan, Mark Olchanyi, Devang Sehgal, Maxina Sheft, Amelia Strom, Berkin Bilgic, Brian Edlow, Elizabeth M. C. Hillman, Meher R. Juttukonda, Laura Lewis, Shahin Nasr, Aapo Nummenmaa, Jonathan R. Polimeni, Roger B. H. Tootell, Lawrence L. Wald, Hui Wang, Anastasia Yendiki, Susie Y. Huang, Bruce R. Rosen, Randy L. Gollub

**Affiliations:** 1 Harvard–MIT Division of Health Sciences and Technology, Cambridge, MA USA; 2 Athinoula A. Martinos Center for Biomedical Imaging, Charlestown, MA USA; 3 Harvard Biophysics Graduate Program, Cambridge, MA USA; 4 Massachusetts Institute of Technology, Cambridge, MA USA; 5 Massachusetts General Hospital, Boston, MA USA; 6 Harvard Medical School, Boston, MA USA; 7 Mortimer B. Zuckerman Mind Brain Behavior Institute, Columbia University, New York, NY USA; 8 Department of Biomedical Engineering, Columbia University, New York, NY USA; 9 Department of Radiology, Columbia University, New York, NY USA

**Keywords:** Neuroimaging, Mesoscale, fMRI, Diffusion, TMS, Optical imaging

## Abstract

Advances in the spatiotemporal resolution and field-of-view of neuroimaging tools are driving mesoscale studies for translational neuroscience. On October 10, 2023, the Center for Mesoscale Mapping (CMM) at the Massachusetts General Hospital (MGH) Athinoula A. Martinos Center for Biomedical Imaging and the Massachusetts Institute of Technology (MIT) Health Sciences Technology based Neuroimaging Training Program (NTP) hosted a symposium exploring the state-of-the-art in this rapidly growing area of research. “Mesoscale Brain Mapping: Bridging Scales and Modalities in Neuroimaging” brought together researchers who use a broad range of imaging techniques to study brain structure and function at the convergence of the microscopic and macroscopic scales. The day-long event centered on areas in which the CMM has established expertise, including the development of emerging technologies and their application to clinical translational needs and basic neuroscience questions. The in-person symposium welcomed more than 150 attendees, including 57 faculty members, 61 postdoctoral fellows, 35 students, and four industry professionals, who represented institutions at the local, regional, and international levels. The symposium also served the training goals of both the CMM and the NTP. The event content, organization, and format were planned collaboratively by the faculty and trainees. Many CMM faculty presented or participated in a panel discussion, thus contributing to the dissemination of both the technologies they have developed under the auspices of the CMM and the findings they have obtained using those technologies. NTP trainees who benefited from the symposium included those who helped to organize the symposium and/or presented posters and gave “flash” oral presentations. In addition to gaining experience from presenting their work, they had opportunities throughout the day to engage in one-on-one discussions with visiting scientists and other faculty, potentially opening the door to future collaborations. The symposium presentations provided a deep exploration of the many technological advances enabling progress in structural and functional mesoscale brain imaging. Finally, students worked closely with the presenting faculty to develop this report summarizing the content of the symposium and putting it in the broader context of the current state of the field to share with the scientific community. We note that the references cited here include conference abstracts corresponding to the symposium poster presentations.

## Introduction

**Fig. 1:**
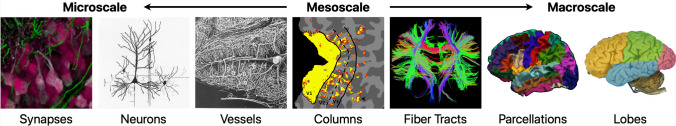
Hierarchical scales of neuroanatomical topology are imaged by various modalities reviewed herein. Partially adapted from Nasr et al., 2016

The brain is a complex network of hierarchically organized and interconnected elements and spans multiple scales from individual synapses on a neuron to large functionally connected regions (Fig. 1). The central hypothesis of neuroscience is that brain function is a global property which emerges over time from the integrated interactions of subunits across scales of neural topology. Constrained by the technologies available in the past, neuroimaging—the primary tool driving studies of the spatiotemporal patterns of connections and interactions within the brain—has traditionally explored this hypothesis from two extremes of neural organization: the microscale (individual neurons and synapses) and the macroscale (whole brain regions). While decades of knowledge have been gained from investigations at the ends of this scale, the historical inability to image neuroanatomical structures in the middle scales of neural organization (the so-called mesoscale) has limited our understanding of processes, disorders, and diseases. The trade-off between resolution and field-of-view (FOV) forced a choice—to either observe a few individual neurons or acquire low-resolution images of the whole brain. Now that the resolution of the large-FOV techniques and the FOV of methods with microscopic resolution are both increasing, we can image on the mesoscale.

Advances in multiple different neuroimaging modalities were presented at the symposium, with a particular focus on novel magnetic resonance imaging (MRI) and optical imaging methods that have made it possible to acquire mesoscale neuroimaging data with higher fidelity and throughput. Speakers presented work on state-of-the-art MRI methods—spanning novel MRI instrumentation, acquisition methods, signal processing, optimization, and machine learning developments—to image the brain at unprecedented spatial and temporal scales. This article reviews the importance of many of these methodological breakthroughs, and highlights discoveries that have resulted from interrogation of the brain at the mesoscale. Specific focus areas included functional MRI (fMRI), diffusion MRI (dMRI), quantitative MRI (qMRI), transcranial magnetic stimulation (TMS), and optical microscopy.

First, we present advances discussed in the symposium in Blood Oxygenation Level Dependent (BOLD) fMRI spatial resolution which have allowed researchers to more effectively investigate the biological resolution of the hemodynamic changes that occur in response to neural activity. Speakers grappled with a key question: what is the biological spatial resolution of fMRI? Additional presentations focused on methods to increase BOLD fMRI speed to match the temporal resolution of the fastest hemodynamic response to rapid neuronal events, harnessing mesoscale resolution to resolve details previously blurred out in less-resolved BOLD response maps. We then proceed to review novel high-resolution MRI and novel reconstruction techniques presented by speakers that have enabled new insights into brain connectivity using dMRI-based tractography at the mesoscale. Included were presentations of the Martinos Center’s pioneering work in the development of ultra-high gradient strength scanners: both the Connectome 1.0 and 2.0 (Fan et al., 2022; Huang et al., 2021). The latter can reach a maximum gradient strength of 500mT/m with maximum slew rate of 600 T/m/s—the highest ever attained for in vivo human imaging. We also review presentations on state-of-the-art qMRI, advanced accelerated acquisition and reconstruction techniques that permit efficient, high-resolution estimates of brain tissue properties while minimizing acquisition time to enhance clinical translatability. Developments in instrumentation have shown critical improvements in the safety and efficacy of ultra-high field MRI, resulting in more widespread application of state-of-the-art high field systems (Budinger et al., 2016; Polimeni & Wald, 2018). Such improvements have been locally demonstrated on two ultra-high magnetic field MRI (UHF ≥ 7 T) 7 T human scanners and a custom-developed 14 T pre-clinical scanner at the Martinos Center to improve imaging sensitivity and resolution. Therapeutic applications are also reviewed: symposium speakers discussed how increasing the sensitivity, resolution, and clinical viability of fMRI and dMRI has underscored their role in interventional applications, such as transcranial magnetic stimulation (TMS). Development plans were presented for a novel hybrid head coil array, allowing for concurrent multifocal TMS with high-resolution fMRI and dMRI of the whole brain.

Finally, presenters reviewed recent developments in optical mesoscale imaging using animal models. Advances in acquisition, FOV, and data processing in optical microscopy techniques such as wide-field optical mapping (WFOM) and Swept Confocally Aligned Planar Excitation (SCAPE) microscopy have enabled real-time functional imaging studies spanning whole brains with micron to millimeter scales (Bouchard et al., 2015; Voleti et al., 2019; Ma et al., 2016a, 2016b; Shahsavarani et al., 2023). Light sheet microscopy and optical coherence tomography (OCT) have also enabled volumetric imaging with unprecedented spatial resolution that spans the mesoscale and microscale (Wang et al., 2018). These technological strides are working to bridge the gap from the opposite direction – increase the FOV of microscopy techniques to allow whole-brain imaging not just in small animals but in the human brain.

The event content, organization, and format were planned collaboratively by the faculty and trainees. We joined efforts with the full faculty leadership team of Dr. Rosen’s NIBIB funded P41 Center for Mesoscale Mapping for this offering. A retreat was held with a 1:1 ratio of faculty and Neuroimaging Training Program (NTP) trainees to brainstorm ideas for the focus and format of the symposium. Trainees wrote up a brief synopsis of their research which was shared with faculty in advance and discussed with group during the meeting. Thus, the trainees’ own research projects formed the foundation of the symposium content. The retreat was followed by a second meeting to finalize content and presenters who would be of greatest common interest to the scientific community.

During the retreat we developed a format that would enable the greatest learning experience for symposium participants. Trainee experience was prioritized through opportunities to present their own research and engage with the broader scientific community to receive feedback on their projects. We created a coherent program to allow trainees to see how their work fits into the broader context of research in this field. The symposium event started with the “Why” of mesoscale mapping—addressing the clinical translational and basic neuroscience rationale for developing mesoscale capabilities through a series of keynote lectures and a panel discussion. The afternoon focused on the “How” of mesoscale mapping, with lectures exploring technologies either currently available or under development and the ways in which researchers are applying them. Between the morning “How” and afternoon “Why” sessions, symposium attendees presented posters on their current research in this field. Every poster presenter was given a 3-min time slot for a “flash” presentation summarizing their work. The symposium ended with a fireside chat with Martinos Center director Bruce Rosen and Harvard University professor Jeff Lichtman. Both neuroimaging pioneers, Rosen and Lichtman discussed the next frontiers in mesoscale mapping, the opportunities the many new technologies afford, providing trainees with an opportunity to explore potential future directions for their research careers.

This report provides a systematic review of the presented advances in the context of mesoscale neuroimaging (Fig. 1). In some sections, additional background information is provided to better orient the reader to the implications of the newest developments. The reader will note that the material presented is both multimodal and highly multidisciplinary—highlighting MRI, optical imaging, and related advances made using tool sets from neuroscience, physics, biology, and engineering. This broad scope reveals the unique challenges and opportunities associated with mesoscale neuroimaging, a truly multimodal, transdisciplinary research space.

## Functional MRI

The brain maintains a high energy demand but has a relatively low energy storage capacity, necessitating a constant delivery of oxygen- and nutrient-rich blood, supplied through the vascular system. At times of elevated regional metabolic activity (such as neural activation in response to a stimulus or task), physiological mechanisms exist to increase blood flow to specific brain regions, allowing for increased nutrient delivery and other metabolic functions (Yablonskiy et al., 2000). The term “flow-metabolism coupling” (alternatively referred to as “neurovascular coupling (NVC)”) describes these local modulations in hemodynamics to match increases and decreases in brain activity.

BOLD imaging is a specific type of fMRI acquisition that enables the observation of brain activity through the detection of hemodynamic changes through a complex weighting of blood flow, volume, and changes in the local concentration of deoxyhemoglobin. In BOLD-weighted MRI acquisitions, deoxygenated hemoglobin (HbR) decreases the signal from tissues, compared to oxygenated hemoglobin, due to its paramagnetic nature (Bandettini et al., 1992; Kwong et al., 1992; Ogawa et al., 1992; Thulborn et al., 1982). Specifically, a ‘positive BOLD’ response corresponds to a decrease in HbR, which is caused by the net decrease in tissue blood oxygenation, despite elevations in blood volume and CMRO2. To analyze BOLD imaging data and characterize activation-based hemodynamic changes, a mathematical model of the hemodynamic response (HDR) to brief neuronal activity, defined as the hemodynamic response function (HRF), is employed. Early efforts to introduce BOLD fMRI as a dynamic indication of neuronal activity were met with some skepticism in parts of the neuroscientific community, as the extant means of measuring brain activity at the time was sequential PET measures of blood flow. Detailed biophysical and electrophysiological experiments during the mid and later 1990’s established that fMRI signal indeed reflected NVC (see e.g. Logothetis et al., 2001). With this improved understanding of the physiological basis of fMRI signals better established, the rate of adoption of BOLD fMRI rapidly advanced in popularity as a surrogate for noninvasively measuring brain activation in vivo (Rosen & Savoy, 2012).

### Does Voxel Size or Biological Resolution Limit the Accuracy of fMRI?

A fundamental question in fMRI, central to our understanding and interpretation of the BOLD response, remains largely unresolved: is the accuracy of fMRI today limited by the imaging voxel size, or the biological resolution of the neurovascular response? This question was explored by Dr. Jonathan Polimeni in the opening session of the symposium. Functional hyperemia (increases in cerebral blood flow as a result of neuronal activation) and by extension fMRI was historically considered to be limited to the macroscale (2–5 mm) (see reviews by (Logothetis & Wandell, 2004; Polimeni & Wald, 2018) based on the hypothesis that smooth muscle and sphincter-driven regulation of arterial vessel diameters occurred upstream from the microvasculature. However, continuing evidence (Poplawsky et al., 2015; Hall et al., 2014; Hill et al., 2015; Longden et al., 2017; Peppiatt et al., 2006; Poplawsky et al., 2019; Schaffer et al., 2023) suggests that this mechanism of control may occur on a biological scale far below the 2 mm limit. For example, coupling mechanisms between capillary regions, which can sense neuronal activity via extracellular potassium (K +), and upstream arterioles may directly contribute to flow regulation (Chen et al., 2014; Longden et al., 2017; Schaeffer & Iadecola, 2021). It has been suggested that pericytes in rats may regulate cerebral blood flow by modulating individual capillary diameters, where changes in blood flow may be coupled most strongly to functional activity, shown in both ex vivo (Peppiatt et al., 2006) and in vivo (Hall et al., 2014) experiments. Another study (Hill et al., 2015) debates the mechanism of control, suggesting that smooth muscle cells, not pericytes, are responsible for contraction, but affirmed that vessel contraction occurs in mice cerebral micro vessels below 10 µm in diameter. More recently, the essential role of sphincters located at the penetrating arteriole (PA)/first-order cerebral capillary junction in maintaining cerebral perfusion in mice was observed (Grubb et al., 2020) (average diameters of the PA, precapillary sphincter, and first-order capillary were 11.4 ± 0.6, 3.4 ± 0.2, and 5.3 ± 0.2 µm respectively). While the mechanisms of NVC in cerebral microvasculature are not yet exhaustively understood, current research indicates that direct flow control occurs at least at the level of precapillary arterioles (Schaeffer & Iadecola, 2021) (which have diameters < 30 µm in diameter in human vasculature (Grubb et al., 2020))—breaching the mesoscale. Therefore, as our understanding of microvascular physiology continues to advance, so too has the need for higher spatial resolution functional imaging.

Hemodynamics-based “intrinsic signal” optical recording of the oxyhemoglobin in the macaque visual cortex is an example of a functional neuroimaging approach which illustrates the high-biological resolution coupling (well below 1 mm) of the vascular response to neuronal activation (Lu & Roe, 2007; Lu et al., 2017). This work successfully visualized hallmark features in functional architecture, including the iso-orientation domains and ocular dominance (or “zebra stripe”) columnar patterns in visual cortex (e.g. see example in Fig. 1 (Nasr et al., 2016). In theory, fMRI measurements should reflect structures of a similar biological resolution. Successful functional imaging must exhibit both high sensitivity (or signal-to-noise ratio) and specificity (correlation between the measured signal and neuronal activity). A previous study from Dr. Polimeni’s group (Polimeni et al., 2010) demonstrates an effective approach towards achieving higher resolution, higher specificity BOLD imaging. In this study, the subjects were first exposed to a visual stimulus pattern designed to produce a letter “M”-shaped fMRI activation map in their primary visual cortical area, or V1, utilizing the well-known retinotopic mapping in the human visual cortex. An UHF (7 T) high-resolution gradient-echo (GE) echo planar imaging (EPI) sequence was employed to obtain BOLD maps at 1 mm resolution. fMRI sampling has traditionally included the pial surface, which can cause the signal to be dominated by the large pial veins (Boxerman et al., 1995). However, flow changes in large arterial and venous vessels are spatially and temporally decoupled from the capillary beds where activation occurs (Duong et al., 2003; Lee et al., 1999; Parkes et al., 2005; Poser & Norris, 2007; Yacoub et al., 2003, 2005; Zhao et al., 2004). Ideally, then, high-specificity fMRI would preferentially sample the capillary bed regions where hemodynamic changes most closely reflect the neural activity. This is a concept pioneered by the early use of a perfusion-based fMRI acquisition strategy (Duong et al., 2001), which exhibited lower sensitivity to larger veins and indicated that selective avoidance of non-specific vessels could enable the resolution of submillimeter columnar patterns.

**Fig. 2:**
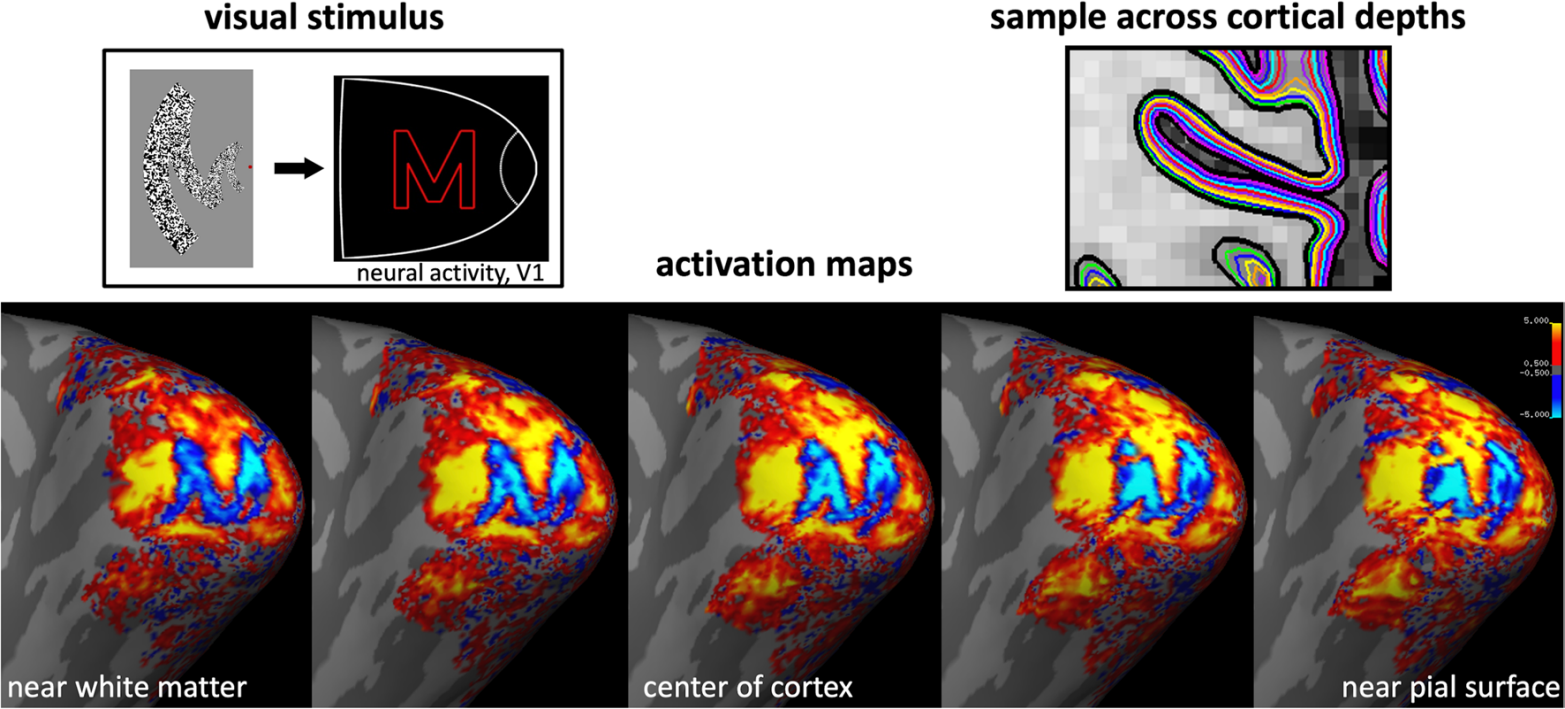
(top left) Visual stimulus and a schematic of the expected resulting visual activity map in V1. (top right) Different cortical sampling depths at each solid-color line. (bottom) Measured BOLD activation maps in the visual cortex of a human subject as a result of the indicated visual stimulus. Reprinted from Polimeni et al., 2010, with permission from Elsevier

Dr. Polimeni’s previous work, highlighted in his presentation, showed how smaller voxel sizes combined with “anatomically-informed sampling” can be used to selectively avoid sampling the pial surfaces using BOLD imaging, improving the spatial localization of the neuronal activation pattern by about 40% (Polimeni et al., 2010). Unfortunately, the sensitivity of the BOLD signal is lowest where the specificity is highest: voxels sampling deep and middle-cerebral cortical layers near to the white matter interface, which predominantly host microvasculature, exhibited approximately half the BOLD signal sensitivity when compared to the pial surface voxels. Nevertheless, by sacrificing a level of sensitivity and selectively sampling the deeper cortical laminae (closest to the white matter), BOLD specificity was increased substantially, and the target “M” pattern emerged in the BOLD activation maps (see Fig. 2). Later work revealed that this use of high-resolution, anatomically-informed, selective sampling revealed ocular dominance columns (ODCs; again, in area V1) and color- and disparity-selective columns (areas V2 and V3) in the human visual cortex, showing patterns similar to those observed with invasive optical imaging by Lu et al. (Lu et al., 2017). Various groups (Dumoulin et al., 2017; Haenelt et al., 2023; Li et al., 2019; Shahin et al., 2016) have now implemented high-resolution, anatomically-informed, selective sampling of the BOLD signal to reveal the columnar patterns in V2 and V3, namely interdigitated stripe systems. Notably, Yacoub et al. (Yacoub et al., 2007) showed that spin-echo (SE) EPI of BOLD signals could also provide higher specificity and clearer depiction of the columnar patterns in V1. Nevertheless, SE EPI suffers from several drawbacks compared to GE EPI: its lower overall sensitivity, increased Specific Absorption Rate (SAR) in the patient, and unwanted GE contamination due to T2* decay at the edges of k-space (Polimeni et al., 2010) make GE EPI BOLD a desirable alternative.

### Additional Applications: Mesoscale fMRI Provides Critical Neurobiological Insights

Mesoscale BOLD fMRI of the visual cortex has been key to providing insights into the nature of visual impairments caused by amblyopia. Amblyopia, colloquially known as a ‘lazy-eye’, is a disorder of neural development which impacts visual acuity in one eye, and thus interocular balance, stereoacuity, and response to high spatial frequency stimuli (McKee et al., 2003). Dr. Shahin Nasr discussed two of the most common causes of amblyopia: anisometropia (asymmetric refraction of the eyes) and strabismus (eye misalignment). Ocular dominance (OD), the tendency to preferentially respond to stimulation from one eye over the other, increases in cases of amblyopia (Conner et al., 2007). This is reflected in the behavior of the V1 area zebra stripes that demonstrate increased neuronal responsiveness to signals from one eye (Hubel & Wiesel, 1962).

**Fig. 3:**
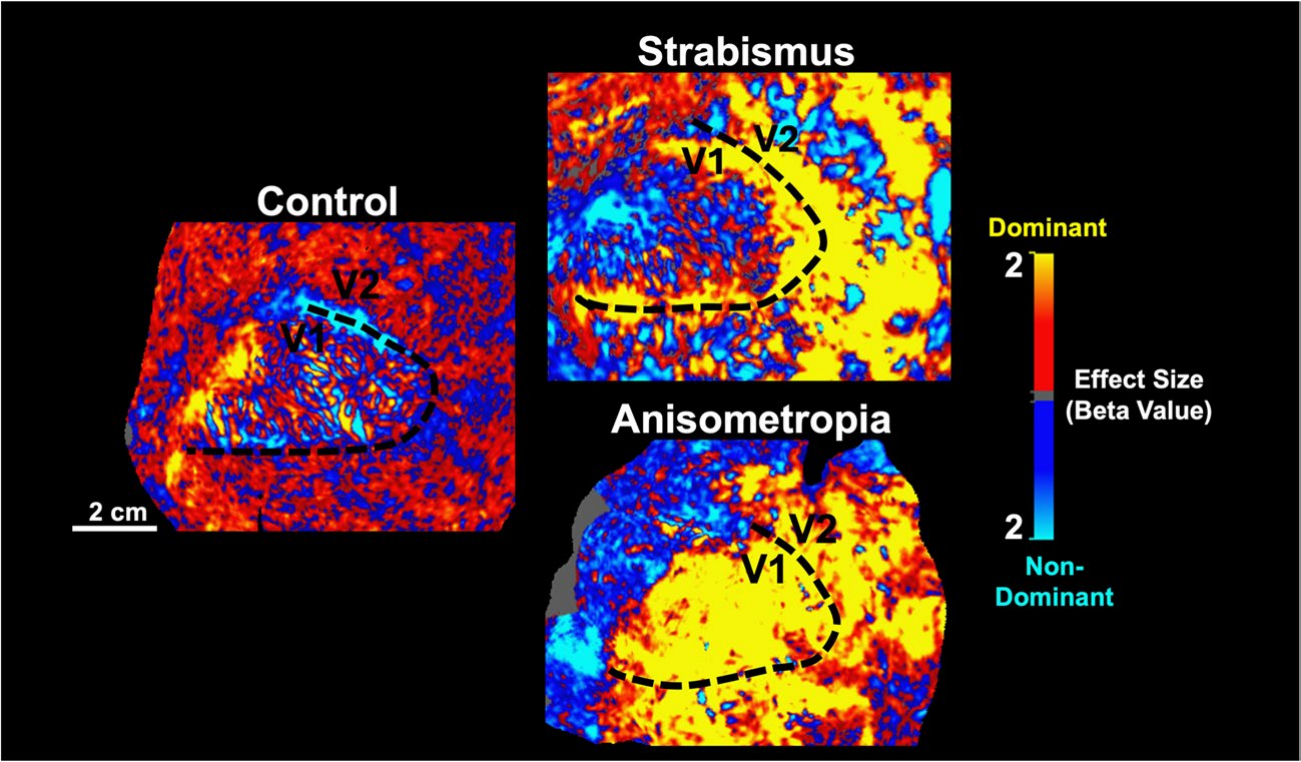
Shown above is the representative activity of OD responses in the superficial depths of the left visual cortex in control (left) and amblyopic participants with strabismus (top right) and anisometropia (bottom right), as imaged with 7 T fMRI. Yellow/red and blue/cyan regions indicate OD responses from a visual stimulus applied to the dominant and non-dominant eyes respectively. Control participants display an OD pattern confined to V1, representative of the known morphology of V1 OD stripes. Participants with both strabismus and anisometropia display OD responses that mostly lack the aforementioned striped pattern, with evoked activity from dominant-eye stimulation extending across both V1 and V2. Adapted from Nasr et al., 2024

Dr. Nasr presented his work using mesoscale fMRI to investigate the ocular dominance response differences in visual cortical areas between healthy and amblyopic individuals (Nasr et al., 2024). His work shows that significant differences exist in the distribution and amplitude of OD responses across multiple layers of the visual cortex in amblyopic compared to healthy participants. The most pronounced increase in OD in amblyopic individuals compared to healthy participants occurred in V1. However, this effect extended to the downstream visual areas, and even V2-V4 (compared to V1) demonstrated stronger correlation between the OD response level and interocular visual acuity difference in these same individuals. Dr. Nasr proceeded to discuss how fMRI can be used to differentiate the impact of amblyopia in anisometropic vs. strabismic participants (Fig. 3). Specifically, there was a greater representation of the dominant eye in V1 in anisometropic individuals as compared to strabismic individuals. Further differences in anisometropic versus strabismic subjects included significantly increased correlation between the OD responses across V1 layers for the former.

Mesoscale fMRI is also advantageous for investigating the relationships between neural organization within specific brain regions that underlie higher-level cognitive processes. For example, Dr. Roger Tootell presented his work investigating columnar organization and neural relationships within the parietal cortex. The inferior parietal cortex is highly involved in the processing of personal space and the perception of intrusions into one's personal space (Tootell et al., 2021). Personal space is roughly defined as the area within a subject’s arm’s reach from oneself, with an average distance of approximately 60-100 cm. Although the absolute size of a subject’s personal space is variable across subjects, this distance is generally consistent for each subject and over time (Tootell et al., 2021). Intrusion into an individual’s personal space can provoke feelings of being threatened and discomfort in the subject, and studies have linked the neuroanatomical origin of this response to the parietal cortex in monkeys (Tootell et al., 2022).

The association between circuits within the parietal cortex and the regulation of personal space indicates the involvement of the parietal cortex in defensive behavior. Tootell and colleagues used high-resolution (1.1 mm isotropic) BOLD imaging via ultra-high field (7 T) MRI to investigate the response of parietal cortex to defensive behavior associated with the increase in discomfort caused by approaching visual stimuli. During the imaging procedure, a visual display showed a person approaching or withdrawing over 15 s intervals. The BOLD data results demonstrated two distinct categories of distance encoding in two corresponding columns in the parietal cortex. The parietal cortex has two types of columns (P and D), which are systematically interdigitated (or non-overlapping) relative to each other. The distinct nature of these columns and systematic non-overlap suggests a neural relationship between the two (Tootell et al., 2022). P columns demonstrated responses to images of a face (both moving and stationary) at virtual distances that were within each subject’s personal space boundary. These columns did not generate responses to images of a face that were farther than this boundary. In the majority of the P columns the amplitudes of the BOLD response increased monotonically and nonlinearly as the proximity of the image increased. In the remaining P columns, the BOLD response decreased as image proximity increased. In contrast, the D columns responded to disparity-based, distance cues from random dot stimuli. Similar columns that respond to disparity-based visual cues are known to exist in the occipital cortex (Nasr & Tootell, 2018). This study suggests that the interdigitated columnar structures in the parietal cortex communicate to transform visual signals (representing distance from other individuals) to sensory information and body-centered mapping (Tootell et al., 2022). Accurate characterization of BOLD response within P and D columns in the parietal cortex was enabled by mesoscale BOLD imaging.

### Insights from First Principle BOLD Modeling with Vascular Networks

In his presentation, Dr. Polimeni explored in some depth how the BOLD fMRI signal reflects the magnetic field changes due to a vascular response from an ensemble of vessels contained within a single imaging voxel, each reflecting an interplay between oxygenation, diameter, and flow changes corresponding to neural activation. In the search for a possible biological resolution limit in BOLD fMRI, separating the BOLD signal contributions from individual vessels may yield unique insights. In vivo microscopy can achieve imaging resolution on the scale of single capillary vessels but is hindered by its invasive nature, shallow penetration depth, and inability to isolate and measure the effects of oxygen, flow, and volume simultaneously. Realistic Vascular Anatomical Network (VAN) models based on reconstructed vessels from these images, first introduced by Silvie Lorthois (Lorthois et al., 2011) opened the door for using first principles of biophysics to estimate these individual hemodynamic properties in each vessel. Gagnon et al. (Gagnon et al., 2015) then expanded these simulations, combined with models of functional hyperemia, to dynamic VANs covering an entire voxel (over 1,000 vessel segments). They also integrated a virtual MRI simulation to estimate the resulting BOLD signal. This MRI-based approach may be able to provide a bridge between fMRI and individual micro-vessels when investigating the biological resolution of the BOLD response.

**Fig. 4:**
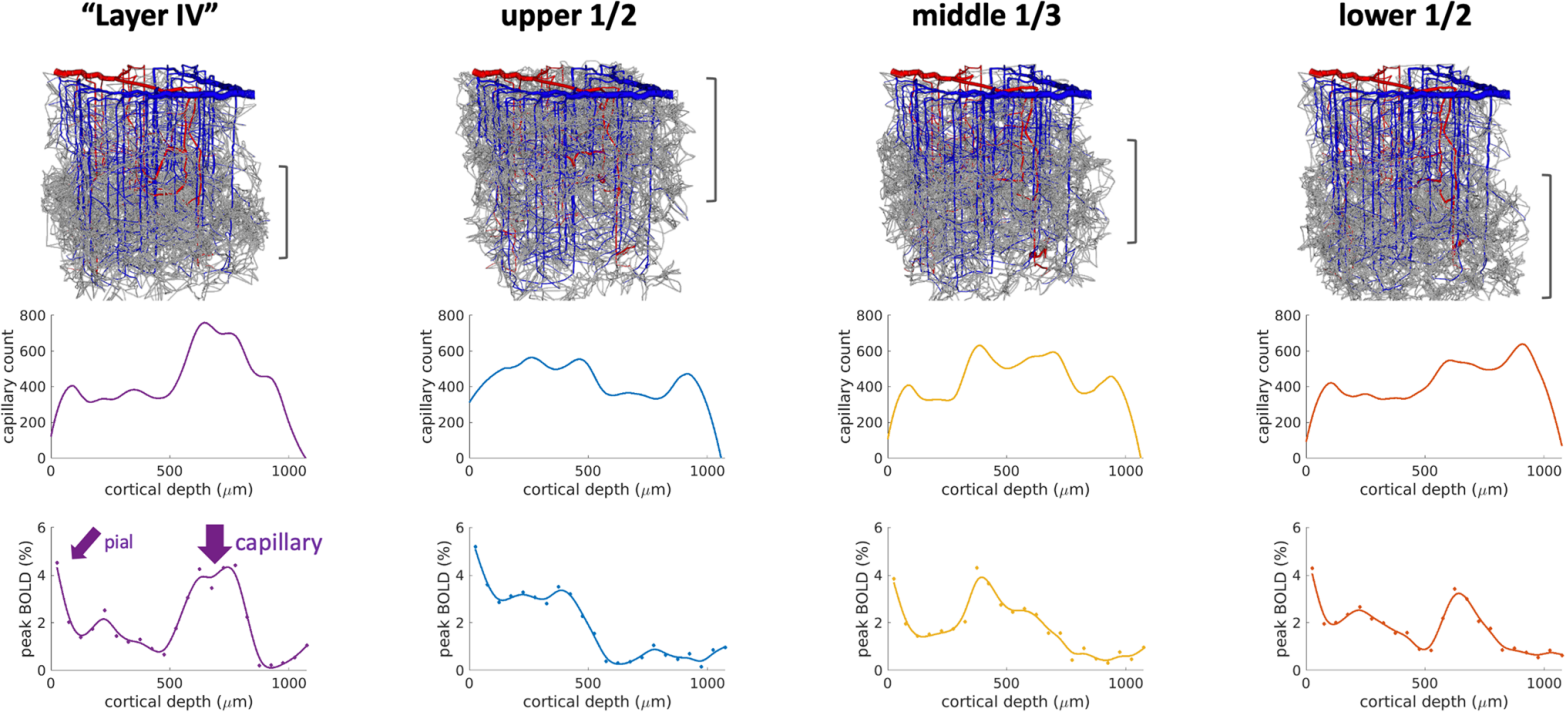
Initial simulation results demonstrating potential microvascular biases in BOLD fMRI in response to excitatory input in cortex. Synthetic vascular networks were generated to imitate cortical mouse vasculature. Simulated peak BOLD signal percentage is plotted as a function of cortical depth (bottom row). Capillary count as a function of cortical depth (middle row) is plotted beneath the corresponding VAN model visualization (top row). Column titles reflect the location of maximum capillary density for each model. Note that results are preliminary and further investigation and validation are ongoing. Adapted from Hartung et al., 2022

VAN models are also being synthetically developed to imitate both mouse and human cerebral vasculature (Hartung et al., 2021a, b; Linninger et al., 2013, 2019; Park & Payne, 2013). These synthetically developed models have enabled researchers to increase VAN size by more than two orders of magnitude, enough to cover the range of typically acquired larger voxels and greater cortical depths (Linninger et al., 2019). Simultaneous work has developed mathematical models and computational algorithms capable of handling such large VANs (Hartung et al., 2018, 2021a). These models have been recently used to compare the impact of vascular anatomical features on the BOLD response, such as local microvascular density, the response spread from individual arteriolar stimulation, and the impact of species differences between mice and humans (Hartung et al., 2022). For example, preliminary results from ongoing work suggest that, in addition to macrovascular biases (such as those induced by larger pial veins, as noted above in the example shown in Fig. 2), there exist microvascular biases in the BOLD signal arising from features in the local vasculature (see Fig. 4). It is well established that areas of increased vascular density are reflective of greater neural activity, where the local metabolic needs regularly demand increased nutrient delivery (Tsai et al., 2009; Weber et al., 2008; Wu et al., 2022). Further work, potentially including both VAN model simulations and higher resolution BOLD fMRI, will be essential to elucidate the relationship between microvasculature and the BOLD response and derive the most accurate interpretation of high-resolution fMRI data.

While mouse-derived VAN models have provided relevant insights, they are not a perfect analog for human neurovascular behavior. Work by Lambers et al. (Lambers et al., 2020) demonstrated that mice exhibit a faster, sharper BOLD response (maximum around 2.8 s) compared to humans (maximum around 5 s), potentially as a result of larger vessel contributions and faster flow velocities compared to humans (de Zwart et al., 2005; Silva et al., 2007). Hartung et al. recently used VAN modeling to point to a possible geometric explanation for this delay using synthetic human VANs (Hartung et al., 2022). However, human VAN fMRI modeling is currently hindered by limited access to the complete cerebrovascular anatomy, including micro-vessel geometry, location, and flow/oxygenation dynamics.

Exploitation of the longer T1 of blood at ultra-high fields is enabling recent advances in geometrically accurate, high-resolution vascular imaging in the brain. A new TOF MR angiography method recently published by Bollmann et al. (Bollmann et al., 2022) revealed full-brain arterial and venous networks with resolution down to 160 μm (isotropic) in a human subject. Initial results from Wang et al. (Wang et al., 2024) demonstrate successful in vivo imaging of intracortical vasculature at using MRI with vessel maps at 64 μm × 64 μm × 1 mm voxel resolution achieved in a non-human primate (macaque). Mesoscale fMRI may enable the identification of potential single-vessel scale contributions to human fMRI that were previously impossible to decipher. Nevertheless, the necessary imaging resolution for reconstructing realistic VAN models at the capillary scale (~ 5–10 μm) is still unattainable using noninvasive methods. Noninvasive single-vessel measurements are currently being developed to measure quantitative changes in diameter and flow in humans with ever-increasing resolution (Hu et al., 2024; Proulx et al., 2024; Varadarajan et al., 2023). In a symposium flash talk, Dr. Divya Varadarajan illustrated her work using single-vessel fMRI in humans to identify potential arterial contributions to the BOLD response. Using ultra-high-field (7 T) MRI, high-resolution, arterial-venous structural images (0.25 × 0.25 × 1.2 mm^3^) and multi-echo fMRI data (0.5 × 0.5 × 1.2 mm^3^)—all at the mesoscale—showed BOLD-weighted changes in individual pial and diving arteries. These results suggest that arterial contributions to the BOLD signal may be non-negligible in some cases.

While the ultimate spatial biological resolution of the neurovascular response is still under investigation, current evidence suggests that it is at least in the mesoscale range (< 1000 µm). Therefore, continued and directed efforts towards the development of higher spatial resolution imaging tools – including acquisition, reconstruction, analysis, and image-informed modeling – remain essential in the search to better understand function and connectivity on the appropriate scale.

### Improving Temporal and Spatial Resolution of BOLD fMRI

To fully capture the dynamism of sensory and cognitive processes, which often unfold on sub-second timescales, high spatial granularity in fMRI needs to be complemented by enhanced temporal resolution. Fast fMRI can observe the fine architecture of the brain and rapid neural activity in near-real-time but presents technical challenges: it requires rapid MRI sequences, and while high spatial resolution unlocks observation of small structures and networks, it can also reduce the signal-to-noise ratio (SNR). However, advances in MRI instrumentation and signal processing methods are beginning to enable fast fMRI studies.

**Fig. 5:**
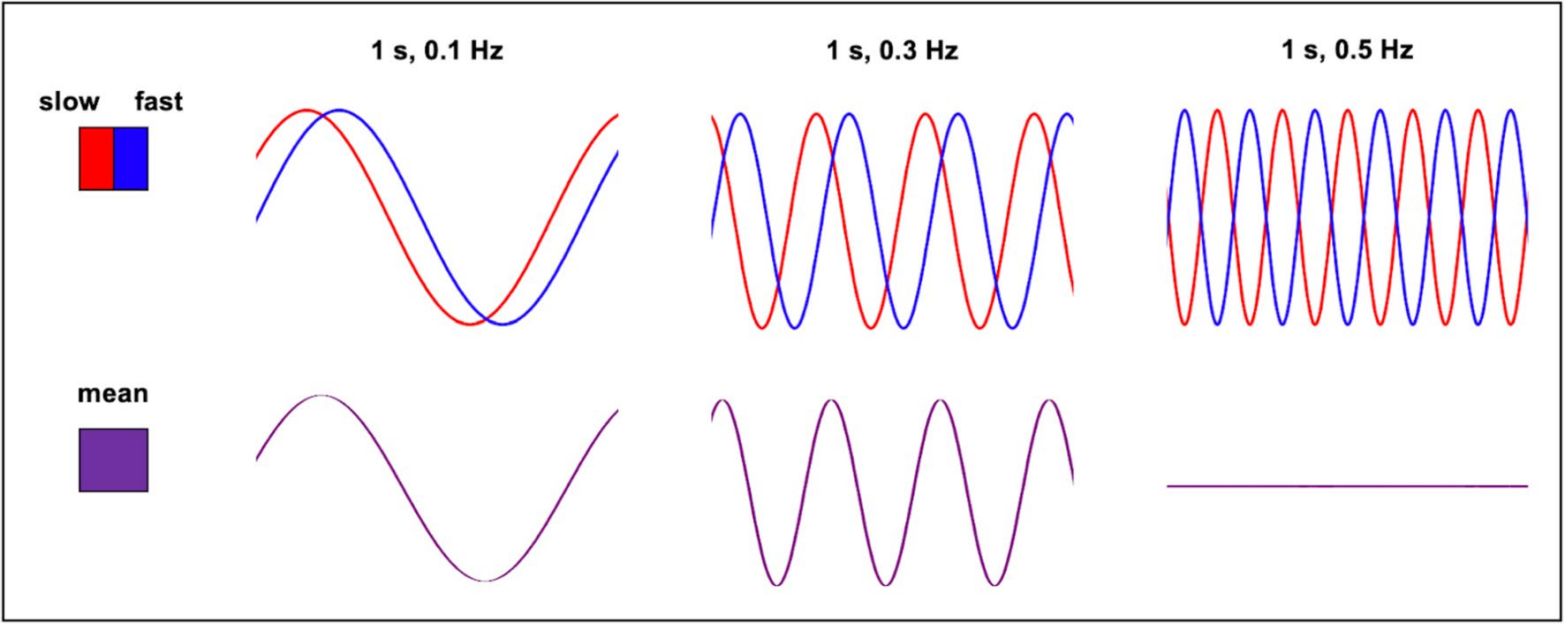
An illustration of how low spatial resolution can lead to loss of signal in rapid neuronal oscillations. Small voxels (red and blue) with small intrinsic differences in hemodynamic response speed, if unresolved due to insufficient spatial resolution, can interfere destructively and result in a misleading measurement in larger voxels (purple) of reduced BOLD signal at high frequencies. Adapted from Chen et al., 2021

In her keynote symposium presentation, Dr. Laura Lewis reviewed the past decade of her work on UHF fast fMRI. Her work has shown that high spatial resolution is key to high temporal resolution which enables measurement of rapid HDRs previously uncaptured by canonical models (Lewis et al., 2016). To put it simply, adequate spatial imaging resolution is essential to resolving rapid HDRs. A large degree of heterogeneity in intrinsic HDR speed across the brain has been observed in both resting state fluctuations (Bailes et al., 2023) and stimulus-driven activation (Lewis et al., 2018). This means that larger voxels can blur distinct HRF curves together, resulting in a broadened mean HRF that obscures fast changes in BOLD response via destructive interference (Fig. 5) (Chen et al., 2021; Polimeni & Lewis, 2021).

**Fig. 6:**
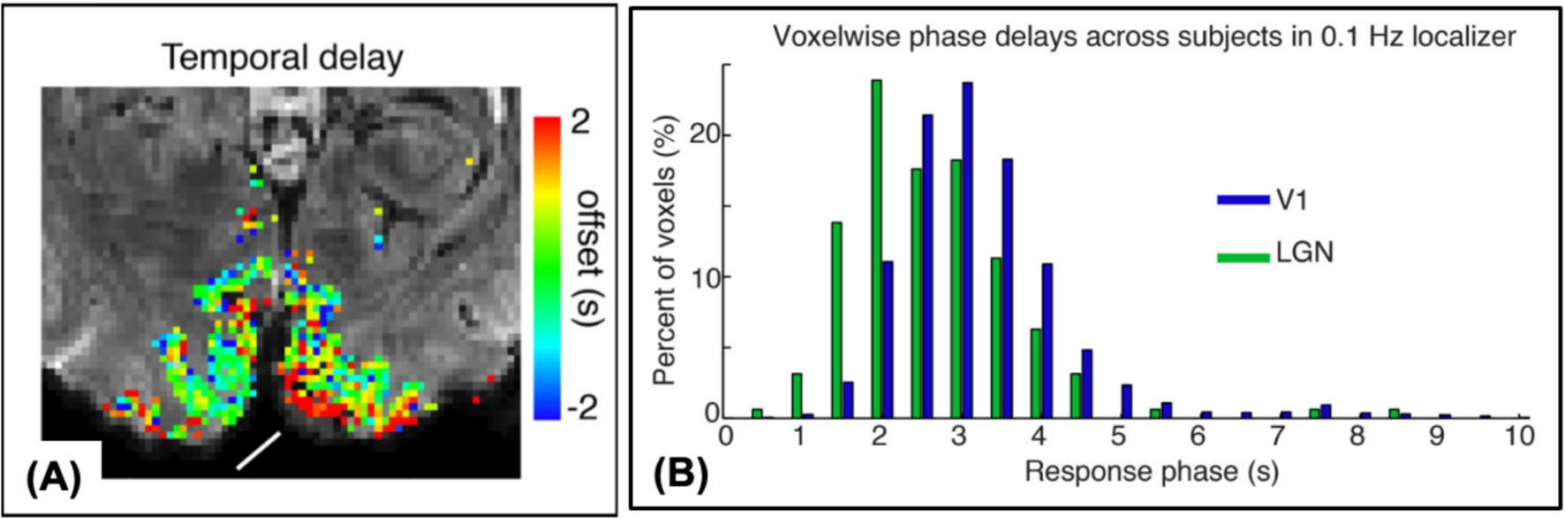
**A** A map of different intrinsic hemodynamic response speeds across small brain regions in the visual cortex. Each voxel has a different temporal delay, color coded to show the offset in seconds. **B** Distribution of the voxel-wise temporal phase delays across subjects in hemodynamic responses in V1 and the lateral geniculate nucleus of the thalamus (LGN) evoked by visual stimulus across subjects. Reprinted from Lewis et al., 2018, with permission from Elsevier

This intrinsic difference in HDR speed across small brain regions also presents an important confound to control for when interpreting fast fMRI signals as a proxy for neuronal activation. Recent studies have successfully characterized differences in hemodynamic response speed across the cortex with a high degree of granularity, which may reflect local differences in vasculature (Gomez et al., 2024; Lewis et al., 2016, 2018) as shown in Fig. 6. Thus, local HDR properties dictate the spatiotemporal resolution required to accurately characterize hemodynamic responses to neural activity with BOLD imaging (Polimeni & Lewis, 2021).

**Fig. 7:**
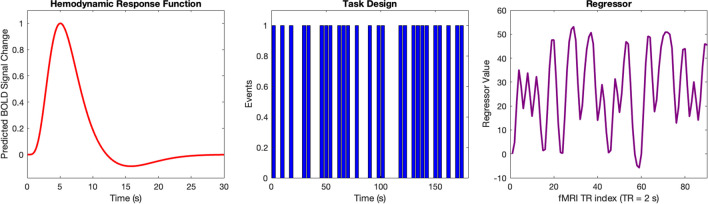
An illustration of typical fMRI data analysis steps. **A** A hemodynamic response function (HRF) is used to model how hemodynamics change in response to sustained neuronal activity. The canonical function peaks 4–6 s after the stimulus and slowly returns to baseline, often with a period of undershoot. **B** The HRF is then convolved with the stimulus paradigm or task-related event design. **C** The convolution analysis produces a regressor for determining what constitutes statistically significant voxel activation corresponding to brain activation in response to the stimulus. Adapted from Chen & Glover, 2015

Interpretation of task-based BOLD fMRI data relies on an accurate mathematical model of the hemodynamic response to the task evoked neural activity (Ogawa et al., 1992). This HRF is typically convolved with the experimental block or event-related paradigm to test for statistically significant activation in voxel time series data, and assumes that the BOLD response is a linear function of changing brain activity, as indicated in Fig. 7 (Chen & Glover, 2015). Canonical HRFs model BOLD response to sustained neuronal activity as peaking 4–6 s after the beginning of stimulus, continuing throughout the stimulus, sometimes having a post-stimulus undershoot, and then slowly returning to baseline. The HDR is generally thought to be intrinsically much slower and less specific than neuronal activity, strongly attenuating high-frequency neural activity, and this approximation predicts little BOLD response at neural oscillations of > 0.2 Hz. However, BOLD response onsets almost immediately in response to increased neuronal demand, and it has been shown with new imaging techniques that a BOLD response to higher frequencies can be observed, evidence that the canonical HRF is only useful for conventional paradigms with long stimulus blocks and rest intervals (Polimeni & Lewis, 2021). Brief or low-intensity stimuli, more analogous to naturalistic neuronal activity, are now known to elicit both faster and larger BOLD responses than can be captured by the linear model (Chen et al., 2021) and some studies indicate that even spontaneous fluctuations may occur on timescales shorter than the ~ 0.1 Hz (Boubela et al., 2013; Chen & Glover, 2015; Jahanian et al., 2019).

**Fig. 8:**
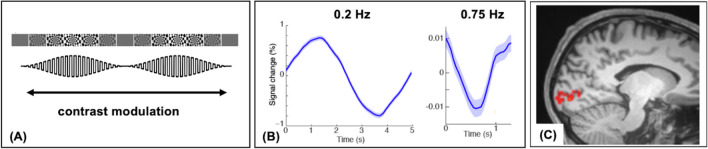
**A** In this experimental paradigm, the contrast of visual stimulus was modulated at several different frequencies, and the BOLD signal response was measured with fast fMRI. **B** Activation corresponding to neuronal oscillations, measured as percent BOLD signal change, was measured on frequencies up to 0.75 Hz in response to the modulated visual stimulus. **C** This signal change was observed in the primary visual cortex, shown here as an activation map of brain voxels. Adapted from Lewis et al., 2016

Resolving the features of these faster, non-canonical HDRs requires MRI acquisition methods with reduced time to repetition (TR). The recent development of simultaneous multi-slice (SMS) acquisition and parallel imaging techniques has enabled fast fMRI using a TR < 400 ms (Jahanian et al., 2019; Setsompop et al., 2012). Additional innovations in ultra-high-field MRI (≥ 7 T) afford higher SNR to boost spatial and temporal resolution (van der Kolk et al., 2013). These advances have enabled whole-brain BOLD measurements with sufficient SNR to resolve HDR at higher frequencies. Prof. Lewis has helped pioneer UHF fast fMRI at the Martinos Center, performing fast fMRI studies at 7 T with a TR of 247 ms (Lewis et al., 2016). In one study, she used BOLD signal changes to measure neuronal activity in response to a contrast-modulated visual stimulus, as shown in Fig. 8 (A). In this paradigm, neural oscillations in the primary visual cortex (V1) and lateral geniculate nucleus of the thalamus were detected up to 0.75 Hz, as shown in Fig. 8B and C. This work shows that hemodynamic responses are often faster than the canonical model suggests and can be resolved with the appropriate tools.

**Fig. 9:**
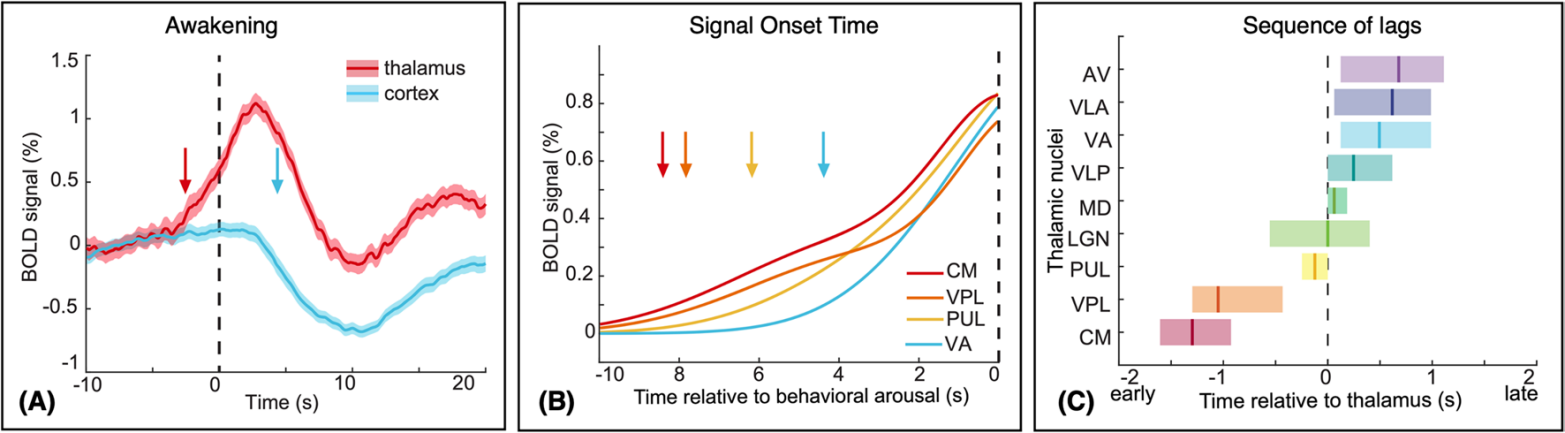
**A** BOLD signal change in the thalamus and whole cortex during awakening. The thalamus begins to activate (red arrow) seconds before behavioral arousal (dashed line) and cortical deactivation (blue arrow). **B** BOLD signal change in thalamic nuclei during awakening (solid lines) and activation onset times (arrows) relative to behavioral arousal (dashed line). **C** Relative timing of thalamic nuclei activation (mean and 95% confidence interval) compared to the timing of the whole thalamus (dashed line) during awakening. Adapted from Setzer et al., 2022 under the Creative Common license (http://creativecommons.org/licenses/by/4.0/). No changes were made to individual figure panels

Recent studies from Prof. Lewis’s lab have also used fast fMRI to show how thalamic activation drives the process of awakening from sleep (Setzer et al., 2022). These methods investigate sub-second dynamics across thalamocortical networks and thalamic nuclei during transitions in behavioral arousal states, uncovering a consistent pattern of activity that precedes the transition from sleep to behavioral arousal. The study mapped out temporally precise signals within the thalamus and cortex during arousal (Fig. 9), demonstrating that a specific sequence of thalamic activity facilitates the transition between behavioral arousal states, with activity in the centromedian (CM) nucleus occurring first, followed by the ventroposterolateral (VPL) nucleus—both activating seconds before the remainder of the thalamus (Fig. 9) (Setzer et al., 2022). These studies show that UHF fast fMRI can be used to investigate neuronal activity on the timescales of cognition.

### Fast fMRI and Future Steps in Functional Neuroimaging

Symposium abstract speakers presented their work on state-of-the-art MRI methods—spanning novel MRI instrumentation, acquisition methods, signal processing, optimization, and machine learning developments—to image the brain at unprecedented spatial and temporal scales. Combining fast fMRI with other imaging technologies, such as Positron Emission Tomography (PET) and electroencephalography (EEG), opens a promising frontier for probing neurochemical dynamics in tandem with neural activation and metabolic processes (Bailes et al., 2023). For example, such tools may allow for a deeper exploration of changes in brain activity and neurotransmitter receptor availability. In a symposium flash talk, Harrison Fisher discussed the trimodal application of fMRI, PET, and EEG to explore changes in activity and D2 dopamine receptor availability across different arousal states. His work illustrated that combining data-driven state change measures from EEG and fMRI with forward kinetic model simulations may help constrain the parameter space and optimize kinetic modeling of complex, naturalistic changes such as sleep. Also under development at the Martinos center is the Human Dynamic NeuroChemical Connectome (HDNCC), a high-spatiotemporal-resolution dedicated brain PET insert integrated with 7 T MRI. This scanner is designed for unprecedented PET sensitivity to enable studies of neurochemical dynamics on similar timescales (and simultaneous) to UHF fMRI (Allen et al., 2024). Accurate spatiotemporal mapping of functional activation is also essential for pinpointing specific areas for targeted interventions, including therapies such as Transcranial Magnetic Stimulation (TMS) as addressed below. These tools will invite unprecedented opportunities in the understanding, diagnosis, and treatment of brain disorders.

## Diffusion MRI

Diffusion MRI is a non-invasive MRI technique used to reconstruct and characterize white matter bundles and gray matter microstructure in the human brain both in vivo and ex vivo (Baliyan et al., 2016; Roebroeck et al., 2019). This method provides insights into clinical and network neuroscience, including neuroanatomy, microstructure in healthy or diseased states, and recovery progression. To generate such maps, dMRI scans acquire images weighted by the direction of the diffusion of water molecules within brain voxels. In white matter, diffusion of water is highly anisotropic due to the coherence of axon membranes and myelin sheaths; water diffuses along the length of axons more readily than it does across the fatty myelin sheath. Tractography algorithms are then used to reconstruct streamlines by following the dominant direction of diffusion across the brain volume. Groups of tractography streamlines that represent specific white matter pathways can be identified based on prior anatomic knowledge. During the symposium, speakers presented ways in which advances in acquisition and analysis techniques for high-resolution dMRI tractography and microstructural modeling have enabled new insights at the mesoscale.

### High Resolution Diffusion MRI

Dr. Gabriel Ramos-Llorden discussed advances in dMRI enabled by the Connectome 1.0 and 2.0 scanners. The Connectome 1.0, equipped with a maximum gradient strength of 300 mT/m, became a key instrument for mapping brain structure and function at multiple scales thanks to higher spatial and angular resolution (Fan et al., 2022). Building on these achievements, Connectome 2.0 was designed to study neural tissue, microstructure, and connectional anatomy with gradient strengths up to 500mT/m, slew rates up to 600 T/m/s, in a 3 T static magnetic field using state-of-the-art RF coil technology for dedicated brain imaging. These advances enable shorter echo times and increased SNR, which allow imaging at higher spatial and/or angular resolution, making the Connectome 2.0 exceptional for mesoscale brain mapping.

Dr. Hansol Lee discussed the application of high-gradient dMRI to understanding age-related changes in gray matter using a technique called soma and neurite density imaging (SANDI) (Palombo et al., 2020). SANDI is a biophysical model of diffusion in both gray and white matter that is used to evaluate cell body size, density, and neurite density from dMRI. Their findings show that high-gradient dMRI provides signal sensitivity to age-related alterations of cortical tissue microstructure in the gray matter which may allow for earlier detection of neurodegeneration. Dr. Florence Chiang presented recent results from the Klawiter lab using SANDI with the Connectome 1.0 to evaluate volume and microstructure changes in thalamic nuclei in multiple sclerosis. Their group found that medial and posterior thalamic nuclei are preferentially affected, suggesting that a cerebrospinal fluid-mediated pathophysiological mechanism is driving thalamic atrophy in multiple sclerosis.

In her keynote presentation, Dr. Anastasia Yendiki discussed how a combination of dMRI acquisition and analysis advances enable dissection of small, deep-brain bundles that are important in psychiatric disease (Maffei et al., 2021). She compared tractography dissections performed on two datasets taken with the Connectome 1.0 scanner: images acquired by the Human Connectome Project at 1.5 mm resolution, and images acquired by Dr. Fuyixue Wang with the g-Slider sequence at 0.76 mm resolution. Both techniques were able to robustly segment and track the fornix, but smaller bundles, such as the stria medularis, mammillo-tegmental tract, mammillo-thalamic tract, and fasciculus retroflexus, could only be resolved with sub-millimeter resolution. This demonstrates the importance of spatial resolution to accurately map brain connectivity. However, the high-resolution data acquired in this study was obtained by scanning a single subject over multiple sessions for a total of 15 h, making this approach to tractography impractical for routine clinical/research use. Yendiki discussed how her group aims to improve access by using a combination of super-resolution methods and algorithmic advances to reconstruct smaller tracts and bundles automatically in more conventionally acquired data.

Clinically acquired dMRI scans are often too low in resolution for reliable white matter pathway reconstruction. These limitations are due to the fact that, when multiple fiber bundles are present in the same voxel, different configurations of these bundles can give rise to the same orientation distribution function. All tractography algorithms make an arbitrary decision on which of the multiple diffusion orientations in a voxel to follow, sometimes leading to anatomically meaningless connections even in high quality dMRI data. However, manually annotated bundles from dMRI scans collected with high b-values and angular resolution (bmax = 10,000 s/mm2, 512 directions) can be used to train an algorithm that can reconstruct the same bundles automatically from scans with lower b-value and angular resolution (b = 1,000 s/mm2, 32–64 directions) (Maffei et al., 2021). Thus, if highly accurate models of white matter pathways are used to inform reconstruction, it is not critical to have data acquired on a scanner with ultra-high gradients or with a long acquisition protocol.

This relies on an algorithm developed by Yendiki and her group, TRActs Constrained by UnderLying Anatomy (TRACULA), which uses a Bayesian framework to incorporate priors based on the anatomical neighborhood of the bundles into tractography reconstruction. When combined with high-quality training data, which can only be acquired on a handful of Connectome-style scanners worldwide, TRACULA is able to reconstruct white-matter bundles with high accuracy from lower-quality dMRI data, allowing the benefits of Connectome-style scanners to reach a wider community. This cannot be achieved by conventional, multi-ROI algorithms for automated dMRI tractography, which rely on post hoc filtering of tractography using ROIs from an atlas (Maffei et al., 2021).

### Ex Vivo Applications of Diffusion MRI

Dr. Brian Edlow presented results of ultra-high-resolution MRI on ex vivo human brain specimens. A 100-µm isotropic resolution multi-echo FLASH (MEF) scan of a single ex vivo brain (Edlow et al., 2019) is now being used as a gold-standard MRI atlas upon which structural and functional (Li et al., 2021) connectivity data can be interpreted with unprecedented neuroanatomic resolution. The 100 µm MRI dataset is also being used to evaluate individual patient responses to invasive and non-invasive neuromodulatory therapies, based on the precise location of brain stimulation targets (Hollunder et al., 2024; Oxenford et al., 2022). Translation of subcortical network mapping at the mesoscale level has the potential to improve the accuracy of diagnosis and prognosis for patients with severe brain injury (Fischer & Edlow, 2024). Clinicians can now identify network disconnections that cause coma (Snider et al., 2019) and preserved network connections that facilitate recovery of consciousness (Thengone et al., 2016). Mesoscale subcortical connectivity maps are also being used in clinical trials to guide the development of targeted neuromodulatory therapies for critically ill patients with severe traumatic brain injury (TBI) (Edlow et al., 2020).

**Fig. 10:**
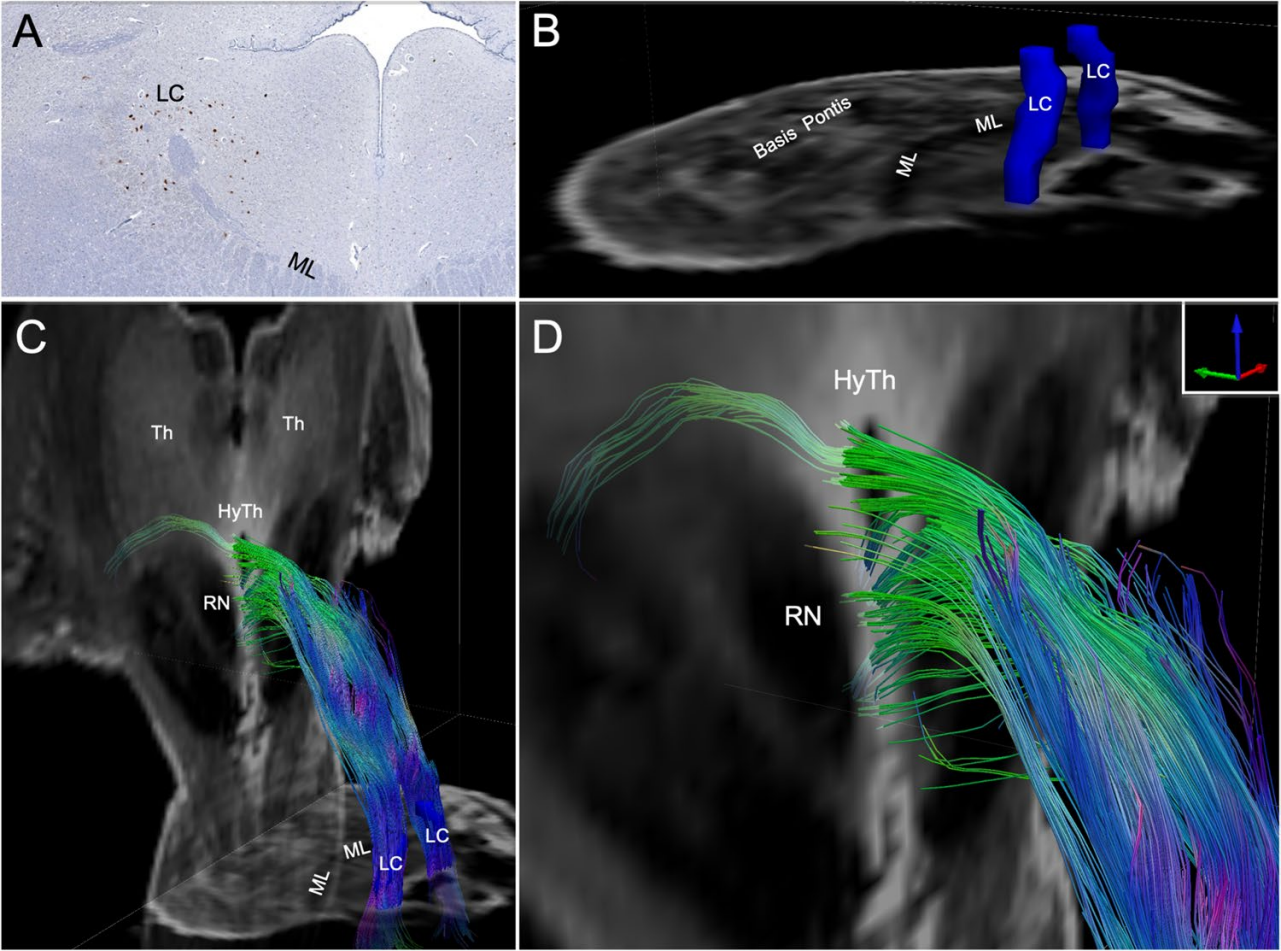
Example of combining histological sectioning with ex vivo DWI. Shown in Panel (**A**) is a tyrosine peroxidase-stained histological section in the pons. This section displays the location and morphology of the noradrenergic cell bodies of the locus coeruleus (LC), a notoriously small brainstem structure that is critical for modulating consciousness. The location of LC cell bodies is then used to accurately localize the LC in corresponding ultra-high-resolution ex vivo DWI (**B**). This histology-guided LC annotation is then used as a seed for tractography analysis of associated white matter connections between the LC and the hypothalamus (**C-D**)

Imaging techniques and processing methods with resolution below 1 mm are critical for studying axonal injury in patients with severe TBI, who have a disconnection syndrome resulting from traumatic shearing of axons. This type of injury often involves microstructural damage to axons that is too small to be detectable with clinical-grade imaging (Maffei et al., 2023; Ng & Lee, 2019). Ex vivo structural and diffusion MRI of the brains of TBI patients provide unparalleled images of white matter microstructure and have revealed numerous structural biomarkers with both diagnostic and prognostic value for TBI (Edlow et al., 2013, 2024). By integrating histopathological data with ex vivo MRI data, a network-based autopsy can be performed, providing insights into brain network disruptions that may have caused neuropsychological symptoms during life (Edlow et al., 2024; Latimer et al., 2023). In a network-based autopsy (Fig. 10), probabilistic tractography from ex vivo diffusion MRI reveals the extent of white matter “disconnection” between regions crucial to sustaining consciousness, such as the ascending arousal network in the brainstem (Snider et al., 2019). Traditional methods of post-mortem assessment of TBI, such as histological staining, typically provide insufficient information from which to infer neuropsychological deficits. Coupling histological findings with high-resolution ex vivo diffusion tractography may advance understanding of how microstructural injury influences network-level changes in disconnection syndromes such as TBI.

Additional ex vivo studies discussed at the symposium include Dr. Ortug’s presentation on the use of ultra-high-resolution (200 um) imaging with a 14 T Bruker scanner to reconstruct white matter tracts in ex vivo 14–15-week fetal brains. These techniques successfully identified unprecedented region-specific neuronal migration pathways in three dimensions in the early second trimester, which may provide a basis for understanding neurodevelopmental disorders, a key step towards early diagnosis and intervention.

### Rapid Acquisition and Reconstruction Techniques for Mesoscale Diffusion MRI

The symposium also explored advances technology permitting improved dMRI, including hardware, acquisition, and image reconstruction techniques. Since slice-by-slice diffusion MRI requires long TRs — which can induce physiological noise and bulk-motion induced phase variations — EPI is often used due to its efficient slice encoding and faster acquisition times (Liao et al., 2023; Mansfield, 1977; Setsompop et al., 2018). However, single-shot EPI is vulnerable to severe susceptibility-induced distortions and T2*- induced voxel blurring at high resolutions (Cho et al., 2024). Multi-shot EPI (msEPI) acquisition seeks to address these concerns by acquiring the entire k-space in multiple excitations to reduce the effective echo spacing (Cho et al., 2024). High-quality msEPI requires combining multi-shot shot data into a single image and correcting for artifacts induced by physiologic motion between multiple scans (Bilgic et al., 2019; Wang et al., 2021). In achieving higher resolution (isotropic sub-millimeter) dMRI, the smaller imaging voxels also have inherently lower SNR (Wang et al., 2021). Thus, mesoscale diffusion MRI is inherently constrained by both scan time and SNR.

One approach to obtain distortion-free diffusion imaging with multi-shot EPI is Blip Up-Down Acquisition (BUDA). In BUDA, two EPI-shots capture complementary segments of k-space, with one employing a positive ky traversal (blip-up) and the other utilizing a negative traversal (blip-down), resulting in opposing distortions. These two shots can be jointly reconstructed with the knowledge of the B0 field map to eliminate distortions (Liao et al., 2021). In the symposium, Dr. Kawin Setsompop presented his group’s work showing how SNR can be improved by combining BUDA with the generalized Slice Dithered Enhanced Resolution (gSlider) technique. This technique improves robustness against shot-to-shot phase corruption through RF-encoding (Setsompop et al., 2018) and uses a circular EPI trajectory for partial Fourier sampling across the blipped-up and blipped-down shots to reduce the echo train length and echo time (Liao et al., 2023). Furthermore, use of a structured low-rank constraint with smooth phase prior (S-LORAKS) (Haldar, 2014; Kim et al., 2017) during reconstruction can also help address shot-to-shot phase variations without resolution loss. Uniting these tools, acquisition with gSlider-BUDA and circular EPI with S-LORAKS reconstruction can achieve 500 mm isotropic dMRI images, though with a low SNR. At slightly lower resolutions (0.72 mm isotropic), ten-diffusion-direction scans are achievable in 9 min (Liao et al., 2023) with improved SNR. However, BUDA can only be used with accelerations of in-plane subsampling factor R of four-fold or lower since it requires interim reconstructions with adequate quality for B0 field map estimation (Jun et al., 2024a). Dr. Berkin Bilgic presented an alternative acquisition scheme for accelerated dMRI named Phase Reversed Interleaved Multi-Echo (PRIME). PRIME enables the creation of a high-quality interim reconstruction which can be used for field map estimation. This is achieved by inserting an additional low-resolution echo into the pulse sequence. The fieldmap estimates are used alongside the first echo data to jointly reconstruct the image using S-LORAKS. Using PRIME with the gSLIDER technique can achieve 1 mm isotropic resolution at eightfold acceleration (R = 4 × 2) with high fidelity (Jun et al., 2024a).

Machine learning methods have also been applied to dMRI to aid in accelerated reconstruction. Network Estimated Artifacts for Temporal Reconstruction (NEATR) is a framework that uses a synergistic combination of a ‘black-box’ machine learning model and a physics-based forward model to reconstruct dMRI images using msEPI (Bilgic et al., 2019). To eliminate the need for training data, a zero-shot, self-supervised learning approach has also been proposed for dMRI, called zero-MIRID (zero-shot self-supervised learning of Multi-shot Image Reconstruction for Improved Diffusion MRI).

### Validation of Diffusion MRI Tractography

Despite advances in both acquisition and analysis techniques, dMRI tractography still faces challenges that were addressed by Dr. Yendiki in her keynote presentation. Tracer injections in non-human primates allow direct visualization of fiber bundles and can thus be used to validate dMRI tractography. In the IronTract challenge that she led, teams had access to ex vivo dMRI data from macaque brains that had previously received tracer injections (Maffei et al., 2021). The teams optimized their dMRI tractography pipelines to maximize their accuracy with respect to the ground truth for one tracer injection site, and then applied the optimized pipeline to another injection site. Outcomes from the IronTract challenge provided recommendations for best practices when analyzing dMRI data and also identified common failure modes across pipelines. Pipelines were often found to fail when confronted with voxels containing large proportions of branching, fanning, and turning fiber bundles. While advances in reconstruction algorithms have improved our ability to resolve crossing fibers, these other fiber configurations are where tractography errors still occur consistently. One proposed solution in the literature is to use microstructural measures to resolve fiber bundles in such areas. However, tracer studies show that fibers from the same injection site do not always remain bundled as they travel through the brain. In some pathways (like the internal capsule or the corpus callosum), axons from the same cortical origin are tightly bundled, while in other pathways (like the uncinate fasciculus) they are more diffuse, and hence interspersed with axons originating from other locations. While atlases can be used to inform the algorithms for reconstructing white-matter pathways from dMRI, they are often limited to larger tracts.

### Safety Considerations in High-Resolution Diffusion MRI

In his presentation, Dr. Wald noted that advances with hardware have led to scanners with increasing magnetic field, gradient strength, and gradient slew rate. However, the fast gradient switching required to enable fast, high-resolution imaging can induce peripheral nerve stimulation (PNS) leading to discomfort and/or pain (Irnich & Schmitt, 1995; Mansfield & Harvey, 1993). Thus, MRI sequences must be designed to minimize incidental neuron activation as further developments are made in MRI hardware (Polimeni & Wald, 2018). Dr. Wald and Dr. Davids have created neurodynamic models to predict the body’s response to electromagnetic fields through a connected nerve atlas. This work subsequently informed the design of the Connectome 2.0 gradients and a novel Siemens head gradient coil “Impulse” for 7 T MRI scanning (Davids et al., 2023) to allow a larger area of their operational parameter space to be used when imaging human subjects. The winding patterns of the coils were adjusted to balance head and body stimulation and increase PNS thresholds by a factor of two relative to the unoptimized design. This coil is being used in studies to further develop ultra-high-field and gradient MRI systems while avoiding PNS (Feinberg et al., 2023; Huang et al., 2021).

## Quantitative MRI

Quantitative MRI (qMRI) involves the acquisition of quantitative tissue parameters of the anatomy being imaged. Examples of quantitative tissue parameters include the T1, T2, and T2* relaxation behavior, magnetization transfer, proton density (PD), and susceptibility maps, flow and diffusion metrics, among others. These parameters reflect the interaction of the water molecules with the tissue components and can thereby provide insight into the local microstructural environment (Weiskopf et al., 2021). Quantitative readouts can allow for easier, potentially automated, tissue characterization (Gulani & Seiberlich, 2020), as well as easier standardization across systems and vendors than currently possible with contrast weighted imaging. qMRI has been widely applied to brain tissue to investigate both normal and pathological processes.

qMRI has important applications in the characterization of brain tissue properties. Using qMRI, tissue properties such as T1, T2, PD, quantitative magnetization transfer (qMT), and quantitative susceptibility maps (QSM) can be calculated at the voxel level, leading to a high degree of spatial specificity. These MR parameters are strongly correlated with different physiologically relevant properties. For example, T1 relaxation time is sensitive to myelin and macromolecular content, PD is sensitive to free water content while the qMT is sensitive to bound water fraction and macromolecular (Carey et al., 2018) content. These multiparametric quantitative maps provide complementary information that has been used to estimate features such as myelin volume fraction per voxel (Fujita et al., 2019), fiber orientation, and to provide data on cortical microstructure (Weiskopf et al., 2021). qMRI has been widely used in studying the effects of aging on cortical microstructure (Callaghan et al., 2014; Carey et al., 2018), as well as demyelination in multiple sclerosis (Mainero et al., 2015).

In qMRI, tissue properties are traditionally estimated by taking multiple scans of the same body part and inferring the parameter values separately (Ehses et al., 2013; Fram et al., 1987). This can be highly inefficient in terms of scan time and lead to suboptimal translatability in research/clinical applications. Holding still for long scan times can be physically challenging, especially for patients in poor physical condition, and longer scan times can increase imaging costs and reduce overall patient throughput. Thus, meaningful deployment of qMRI in both clinical and research settings requires a more efficient imaging approach.

One such acquisition technique is 3D-Quantification using an interleaved Look-Locker acquisition sequence with a T2 preparation pulse (3D-QALAS) (Kvernby et al., 2014). It enables simultaneous acquisition of high resolution T1, T2 and PD information from five measurements in each repetition time (TR) (Fujita et al., 2019; Jun et al., 2023). The two major advantages of 3D QALAS lie at providing high isotropic resolution, and an efficient acquisition. 3D QALAS has been applied in neuroimaging to achieve whole brain T1, T2 and PD scans at 1 mm isotropic resolution within 12 min of scan time (Fujita et al., 2019). Using compressed sensing, an acceleration technique in reconstruction that utilizes the sparsity of an image and allows for subsampling, this can be brought down to under 6 min (Fujita et al., 2019). While several techniques have been proposed for further reducing scan times, fitting of parameter maps in 3D QALAS requires an additional fitting process. This is computationally expensive, requiring the use of a pre-calculated dictionary followed by voxel-by-voxel fitting, preventing online reconstruction (Jun et al., 2023). To address these shortcomings, in his presentation Dr. Bilgic discussed deep learning based approaches such as Self Supervised Learning—QALAS (SSL-QALAS). SSL-QALAS allows for estimation of T1, T2, PD and inversion efficiency quantitative maps rapidly using dictionary-free multiparametric fitting. By using a pre-trained model which could be fine-tuned on the subject’s data, SSL-QALAS was able to estimate whole-brain tissue parameters in 15 min at 1 mm resolution (Jun et al., 2023). Dr. Bilgic described further improvements achieved in 3D QALAS acquisition by the use of zero-shot learning and subspace reconstruction techniques. Zero shot learning techniques do not require fully sampled k-space data, eliminating the need for a training database, while techniques using low rank subspace bases for denoising have enabled high resolution image reconstruction in qMRI (Haldar, 2014). Zero-DeepSub (Jun et al., 2024b) leveraged these ideas to achieve in vivo whole-brain 1 mm isotropic T1, T2 and PD within 2 min of scan time. These improvements are necessary for translation to clinical practice.

MR Fingerprinting (MRF) is an early acquisition and reconstruction technique for qMRI. MRF involves first obtaining a highly under-sampled dataset acquired with randomized TRs and Flip Angles (FAs) that create temporal and spatial incoherence. The parameter maps are then generated voxel-by-voxel by fitting with a pre-computed dictionary, analogous to QALAS. (Ma et al., 2013). 3D acquisition can be accelerated by using a highly undersampled stack-of-spirals approach, where a spiral trajectory is used with uniformly under-sampling partitions in an interleaved fashion (Liao et al., 2017), while reconstruction is boosted with use of the GRAPPA method (Griswold et al., 2002).

While T1 and T2 relaxation maps are the most frequently acquired qMRI maps, there are several other properties of interest for research and clinical use. Later in the symposium, Dr. Albert Jang introduced a novel sequence and modeling strategy named BTX that enables acquisition of simultaneous qMT and QSM, sharing the results of an in vivo study confirming agreement of the parameter maps with published literature. On the reconstruction side, Dr. Wanyu Bian introduced the quantitative Diffusion Model (DiMo), a technique based on Denoising Diffusion Probabilistic Models (DDPMs) for qMRI reconstruction as an alternative to standard fitting procedures. DDPMs represent a generative AI method designed to learn the inverse of the noise generation process (Bian et al., 2023). In DiMo, embeddings derived from prior MRI physics are incorporated into the diffusion model, ensuring data consistency to direct both training and sampling processes. DiMo was demonstrated for T1 quantification and can be generalized to other qMRI reconstruction tasks as well.

The development of highly parallel imaging using receive-coils with a large number of elements provides improved SNR and the ability to mitigate distortion artifacts through parallel imaging reconstructions of undersampled and thus accelerated k-space trajectories (Bodurka et al., 2004; de Zwart et al., 2004; Wiggins et al., 2005). While 32 channel coils have been used frequently with 7 T scanners, recent advances have enabled development of 64 channel (Mareyam et al., 2020) and even 128 channel receiver arrays (Gruber et al., 2023). These allow for a reduction in noise amplification at high accelerations (R = 3 and above) (Mareyam et al., 2020) with the 128-channel array showing an SNR gain of 86.7% at the cortex over corresponding 32 channel systems at R = 5 (Gruber et al., 2023).

Conventional imaging techniques, however, are often unable to make full use of the potential offered by such hardware advances since they do not utilize coil sensitivity information in one of the three dimensions since the readout direction is not undersampled (Bilgic et al., 2015). Thus, synergy between the hardware, acquisition technique, and reconstruction techniques is essential for maximizing efficiency and resolution. Wave-CAIPI is an acquisition and reconstruction technique presented by Dr. Bilgic that seeks to address this issue by undersampling evenly in all three dimensions, utilizing the parallelization thus enabled. It achieves this by concurrently applying sinusoidal gradients across the y and z directions (with a pi/2 phase offset between them) (Bilgic et al., 2015), during readout of each k space line, producing staggered corkscrew trajectories through k-space. This results in well distributed aliasing patterns across all 3 spatial dimensions allowing for highly accelerated volumetric imaging while retaining SNR. The Wave-CAIPI technique can also be used for various imaging sequences to achieve high accelerations through better utilization of the high field strength machines and large number of receiver coils, including the 3D-QALAS method noted above (Cho et al., 2024). Applied to MP-RAGE, Wave-CAIPI achieved an acquisition time of 57 s for 1 mm iso with R = 4 × 3 at 7 T (Polak et al., 2018). When applied to a three-dimensional gradient echo (GRE) sequence, it allows for an efficient acquisition, providing 0.5 mm isotropic resolution at 5:35 min (Bilgic et al., 2015).

Finally, Dr. Bilgic discussed recent efforts to improve MR sequence reproducibility and ease of development with a vendor-agnostic pulse sequence platform deemed “Pulseq” (Layton et al., 2017). Because acquisition and reconstruction are closely linked to hardware for efficient reconstruction, sequence programming has historically been MRI vendor specific. This has resulted in poor reproducibility across sites and limited widespread implementation of new sequence developments. As an open-source software, Pulseq aims to facilitate reproducible sharing of novel acquisition techniques across multiple sites and can facilitate global collaboration and open-source development of (mesoscale) acquisition techniques.

## Incorporating Mesoscale Imaging with TMS

Transcranial Magnetic Stimulation (TMS) is a non-invasive technique to stimulate the brain. A brief, high intensity magnetic field is produced by passing electric current through a coil placed near a subject’s scalp, which can excite or inhibit cortical areas (Hallett, 2007). TMS has been used in research to map brain function and explore the excitability of different regions by studying either physical effects of stimulation, such as motor or sensory effects evoked with stimulation or measuring EMGs in the periphery, or by concurrently recording brain activity by another method such as EEG or fMRI to study the connections between the stimulated area and other brain regions. The fundamental benefit of TMS over other approaches to brain mapping is that it can be used to provide causal information – we can study the sensory and motor consequences of stimulating a specific region of the cortex. We can also perform cleaner behavioral experiments by creating ‘virtual lesions’ that transiently (and reversibly) disrupt the ongoing neural activity in a cortical region (Pascual-Leone et al., 1999). Repetitive TMS (rTMS) also can modulate the underlying brain networks, demonstrating utility for clinical applications (Chail et al., 2018; Cohen et al., 2022; Sonmez et al., 2019).

### Inferring Brain Function from fMRI with TMS

High spatial resolution neuroimaging modalities are currently unable to directly observe brain function. For example, while BOLD fMRI can reveal regions of increased blood flow in the brain correlated with specific neural activities, it cannot answer questions of causality or how these different regions interact. By disrupting brain activity at specific locations, i.e. creating ‘virtual lesions’, and observing the downstream behavioral effects, TMS coupled with high resolution neuroimaging modalities of the whole brain such as fMRI, could be used to generate functional maps of brain connectivity (Friston, 2011). Conversely, TMS can also be used to map functional relationships between regions predicted to be connected by dMRI. For instance, MRI-based tractography does not provide information regarding the nature of the predicted connection between regions, i.e., if the connection is inhibitory or excitatory. Using TMS, this can be tested by sequential stimulation of different nodes. As discussed earlier, while dMRI and advanced tractography algorithms can help infer presence of pathways and tracts between specific locations in the brain, uncertainty and ambiguity in the directionality of these tracts remains. At the symposium, Dr. Aapo Nummenmaa described how TMS can address these concerns and provide the missing causality information in tractography enabling a better understanding of the mechanisms underlying the brain’s response to stimulation.

Stimulating different brain regions using a TMS coil requires physical movement of the TMS coil over the desired location. Improvements in hardware can be used to optimize localization and enable electronic control of the TMS electric field, as well as to improve focality of stimulation pulse. This can be achieved by use of multichannel TMS (mTMS) arrays, enabling targeting of multiple cortical regions with millisecond accuracy. In her presentation, Dr. Navarro de Lara showed how a new type of 3-axis coil design at each channel could be used to provide electronic control of the stimulation “hot spots”. Here, the circular z-element is aligned parallel to the scalp surface with an x/y-element wound on a spherical coil form with interlaced windings (Navarro de Lara et al., 2021). By designing stimulation patterns that make use of the three axes simultaneously, the electric field can be “continuously steered” under the coil array. Another benefit of multi-axis, multichannel systems is that they can offer more spatial flexibility in tailoring the stimulating fields compared to a single-channel TMS system with a fixed spatial stimulation profile. This allows for increased stimulation amplitude and penetration by activating a wider range of coil elements, permitting deeper structure targeting or selectively activation of superficial targets with improved focality. Because the different types of stimulus patterns can be electronically switched rapidly without any physical coil movement, the mTMS systems may open new possibilities to probe the cortical circuits with enhanced spatiotemporal specificity.

Both Drs. Bilgic and Nummenmaa presented work on achieving the next generation causal brain mapping: building a new coil array capable of both stimulation via TMS and high-resolution neuroimaging using fMRI and dMRI. The proposed Array for Reception, Encoding, Shimming, and Stimulation (ARES2) will achieve gradient strengths of up to 1000 mT/m with high slew rates with multi-channel TMS capability. Once complete, the system the team is working on will be capable of interleaved successive stimulation and recording from cortical areas, allowing for unparalleled causal behavioral experiments with sub millimeter imaging resolution. A key challenge in development of concurrent TMS and MRI setups is the trade-off between low SNR acquired or limited spatial coverage in concurrent steps. This occurs because the TMS coils must be placed close to the scalp for stimulation, requiring the use of birdcage RF coils for whole head imaging as a receiver instead of the more recently developed 32 channel/64 channel RF coils in rigid helmets (Navarro de Lara et al., 2023). The TMS coil is placed between the scalp and birdcage coil. Addressing this concern, Navarro De Lara et al. designed a 28-channel receive only RF coil which can be used with mTMS setups in a helmet design. The setup was designed to minimize possible interactions between the TMS and MRI coils, and SNR of 66% and 86% of the 32/20 channel head coil was achieved in the study with a phantom. In a flash presentation at the symposium, Dr. Navarro De Lara also presented her work on building an “RF-EEG Cap” for whole-head concurrent TMS/EEG/fMRI at 3 T at the symposium. A 2-channel prototype using flexible coaxial cable loops were sewn into a cloth cap with EEG electrodes in the middle of each element demonstrated feasibility. A concurrent TMS/EEG/fMRI experiment was conducted using a phantom, with no significant effects induced by presence of the EEG on either the stimulation or SNR of image acquisition.

### Therapeutic Applications: Mesoscale Imaging to Improve TMS

Mesoscale imaging is essential in therapeutic TMS to understand the underlying pathophysiology and design effective treatments. Therapeutic TMS modulates brain activity for the treatment of a range of neuropsychiatric disorders such as major depressive disorder and obsessive–compulsive disorder (Chail et al., 2018; Cohen et al., 2022; Sonmez et al., 2019). Therapeutic TMS inherently requires understanding neural structure and function across a broad range of spatiotemporal scales. Currently, therapeutic TMS targets macroanatomical regions such as the dorsolateral prefrontal cortex (DFPLC). However, the disrupted connectivity that is often observed on the conventional resting-state fMRI scans has underlying pathophysiological changes that potentially occur on the mesoscale. For example, TMS for major depressive disorder targets the DFPLC, an anatomic structure on the order of 8–10 cm3 with stimulation pulses widths on the order of 100–300 µs (Sanches et al., 2009). The TMS pulses are repetitively applied in different patterns (10–20 Hz, Theta burst) over several minutes (rTMS). The biophysical/neuronal effects of individual stimuli occurs on their characteristic temporal and spatial scales: the time-varying magnetic field of the TMS induces an electric field in the body which depolarizes both excitatory and inhibitory cortical neurons in the targeted cortex (Siebner et al., 2022). The stimulation of neuronal elements depends on the relative orientation of the electric field with the axons, and the distribution of the electric field with respect to axonal segments with lower thresholds at bends or terminals. Thus, the effect of TMS is mediated by not only the stimulation parameters like amplitude, pulse width, frequency and orientation of the coil, but also the local geometry and distribution of the neurons being targeted. In addition, this local excitation may spread to connected brain regions via cortico-cortical and other anatomic connections in a highly network and stimulation-location dependent manner (Siebner et al., 2022). Dr. Nummenmaa discussed advances in how specific regions of the brain are targeted in therapeutic TMS. First a high-resolution structural MRI scan of the subject’s brain is used to generate a surface mesh on a head model. This is overlaid with the electric field generated by specified TMS coil position and stimulation parameters. The interactions between the neuronal elements and TMS-induced field can be computed by the Finite Element Method (FEM) (Thielscher et al., 2015) or by the Boundary Element Fast Multipole Method (BEM-FMM) (Makarov et al., 2020). These models can provide a nominal field resolution as high as 40um (Makarov et al., 2020). Performing TMS at the mesoscale requires a combination of advancements in hardware for reducing the size of the stimulated focal area, better computational models to understand what neuronal elements are being stimulated, and concurrent use with high-resolution imaging modalities, like diffusion MRI at the mesoscale to observe the network-level rTMS effects that occur during and after stimulation.

## Optical Imaging

Dr. Elizabeth Hillman in a keynote presentation, and Dr. Hui Wang in a panel presentation, discussed how optical imaging techniques broaden the field of mesoscale mapping by providing alternative methods to analyze brain structure and function. Ground-truth measurements of brain structure and function concurrently at the micro- and mesoscale will aid in the validation of a wide range of human imaging methods. Mapping of the micro-structure of brain pathways using other micro- and meso-scale modalities has become necessary to inform future MRI tractography algorithms by generating better atlases and priors to overcome current limitations with the technique. There are multiple ongoing initiatives and large-scale projects to leverage recent advances in optical microscopy and MRI to capture brain structure and function at unprecedented scale and resolution including the Human brain Optimized Light Sheet (HOLiS) project and the NIH Brain Initiative Center for Large-scale Imaging of Neural Circuits (LINC).

### Wide-Field Optical Mapping

Wide-field optical mapping (WFOM) is a simple imaging technique that uses a camera to observe large areas of exposed rodent cortex (through thinned skull). The field of view can span over 10 mm and is illuminated by visible light of specific wavelengths to measure substances with distinct optical properties such as the oxygenation-dependent endogenous absorption of hemoglobin, or fluorescent calcium or voltage indicators that can report changes in neuronal activity. WFOM can be used to simultaneously measure the functional dynamics of both neuronal activity and hemodynamics across the surface of the brain of an awake, behaving mouse at over 20 Hz, with a spatial resolution of tens of micrometers Ma et al., 2016b). Current state-of-the-art WFOM studies are made possible by higher sampling rates for the camera system, bright and modulatable LEDs for illumination, and improvements in the speed and contrast-to-noise ratios of fluorescent indicators (Chen et al., 2012) to capture higher speed neural activity with higher sensitivity. One important consideration for WFOM studies measuring fluorescent indicators is the potential for hemoglobin absorption dynamics to contaminate neuronal signals. Simultaneous measurement of both neural and hemodynamic signals with WFOM enables removal of these effects, enabling the real-time relationships between neuronal activity and brain hemodynamics to be evaluated and studied (Ma et al., 2016a). Optical investigations of hemodynamics have been instrumental to our understanding of the BOLD signal by describing spatiotemporal features of blood oxygenation changes following neural activity (Devor et al., 2003, 2007; Hillman et al., 2007; Ma et al., 2016a).

Dr. Hillman presented recent work using WFOM to understand the neural underpinnings of dynamic resting-state fMRI signals and their relationship to behavioral state, with direct relevance for a multitude of resting-state fMRI studies (Shahsavarani et al., 2023). The mesoscale experiment was elegantly designed to bridge species and scales, linking commonly observed signals in mice at the cellular level to commonly observed signals in humans at the regional level. Most studies of neural activity and hemodynamics in mice have focused on responses in small regions, and fMRI studies in humans lack information about ground-truth neural activity. The group also investigated any existence of dynamic functional connectivity during different levels of rest, which is commonly reported in human fMRI studies (Preti et al., 2017) but not specifically studied in animal models, which tend to focus on behavioral correlations (Cardin et al., 2020).

**Fig. 11:**
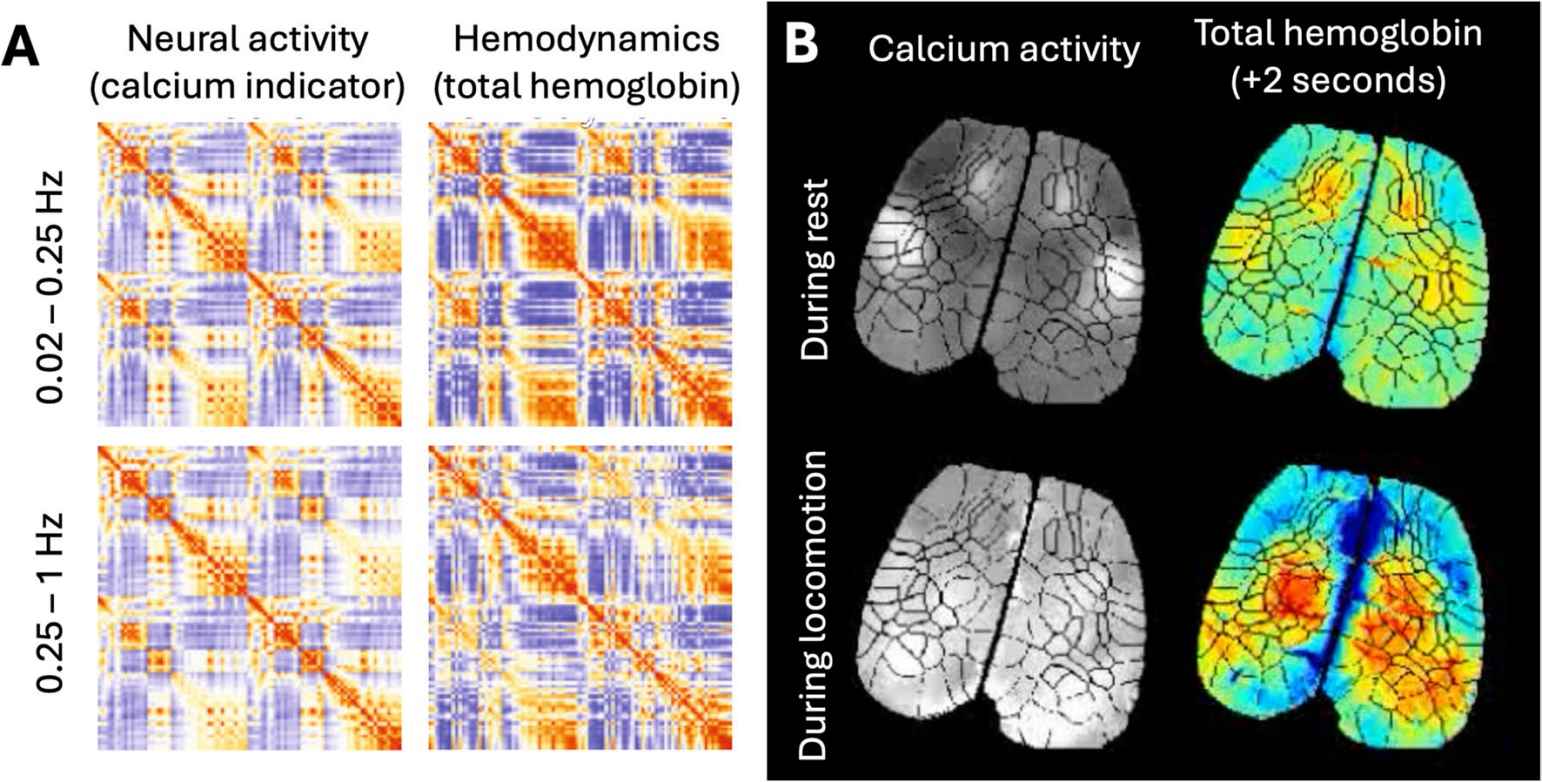
Temporal coupling between neural activity and hemodynamics measured simultaneously with WFOM. **A** Correlation between neural activity and hemodynamics over regions of interest during a 10-s period of sustained rest in low frequency bands. Each row represents a different cortical region. **B** Snapshots of neural activity (left) and hemodynamics (right) captured 2 s later during sustained rest and during locomotion. Adapted from Shahsavarani et al., 2023

Dr. Hillman’s group found that neural activity patterns fluctuate across the mouse cortex over time in a similar manner to what has been observed in resting state fMRI. The hemodynamic patterns observed could be reproduced by applying a temporal low pass filter to the neural data, indicating that hemodynamics are in fact tightly coupled spatially and temporally to patterns of neural activity. Indeed, spatial correlation patterns during rest for neural activity and hemodynamics are highly similar, and periods of increased neural activity are temporally followed by increased hemodynamic activity in the same regions (Fig. 11). Further, they found that hemodynamics are also correlated with high-frequency neural signals, indicating that they reflect broadband neural activity rather than simply low-frequency activity. This is an important point, as hemodynamics are sometimes considered reflective of slow waves of neural activity that are unrelated to high-frequency neural content. The group also observed many hallmarks of resting-state fMRI signals in the neural activity time courses, such as coordinated networks (van den Heuvel & Hulshoff Pol, 2010), dynamic “states” of connectivity seen with a sliding window technique (Preti et al., 2017), and an association of connectivity “state” with the onset of behavior (Greene et al., 2023). Importantly, these same properties were present in the hemodynamic data, providing a validation for the hemodynamic-based signals used in fMRI. The study also provides important context for microscale or cellular-level microscopy studies: the presence of dynamic neural activity during rest indicates that neural activity changes that are not explained by overt behavior may be reflective of dynamic brain states rather than task-independent noise. This experimental design demonstrates how mesoscale imaging in animal models can be leveraged as a validation tool for human imaging. Importantly, these WFOM techniques will be valuable to understand the relationship between hemodynamics and neural activity in the case of impaired neurovascular coupling, since this is the basis for using hemodynamics as a proxy for neural activity (Chen et al., 2014; Hillman, 2014).

### Swept Confocally Aligned Planar Excitation

Swept, Confocally Aligned Planar Excitation (SCAPE) microscopy allows for volumetric imaging with unprecedented temporal resolution and cellular resolution (Bouchard et al., 2015; Patel et al., 2022; Voleti et al., 2019). In her presentation, Dr. Hillman discussed the promises and challenges associated with SCAPE. While most existing optical methods sacrifice imaging speed, spatial resolution, field of view, or sample geometry, limiting their application for in vivo 3D imaging of large samples, SCAPE offers technological advancements in this area at multiple levels. The SCAPE platform is simple and cost-effective as it can be constructed entirely with off-the-shelf components (Voleti et al., 2019). The platform builds on common light-sheet microscopy methods, but uses a single, stationary objective at the sample. The imaging geometry consists of an oblique light sheet that illuminates the sample along the y and z axis, while fluorescent light generated is detected back through the same objective lens, and mapped onto a high-speed camera such that the detected image plane aligns with the oblique sheet plane, providing optical sectioning. The co-aligned oblique illumination and imaging plane are then both swept back and forth over the sample along the x-direction using a single galvanometer mirror. Different configurations of the system can be achieved with replacement of the system’s lenes and camera, attesting to the system’s simplicity and accessibility. However, as datasets acquired with SCAPE can be immensely large given the high spatial resolution, sampling rate, and field of view that are possible, computational advances are required to efficiently store and analyze them.

Dr. Hillman presented her group’s work with the SCAPE platform tracking the neural activity of all cells in the brains of live, behaving Drosophila. They found that neural activity was composed of both broad and local clusters of activity, and described microcircuits that are responsible for specific behaviors (Schaffer et al., 2023). SCAPE microscopy has also been used to track blood flow in a zebrafish heart with cellular resolution by using fluorescent markers of epithelial cells and red blood cells (Voleti et al., 2019). Similar setups may be used for 4D velocimetry and particle tracking in the cerebrovasculature.

For ex vivo structural imaging, high-throughput microscopy methods like SCAPE can be leveraged to their fullest extent when used alongside tissue clearing techniques. While ex vivo microscopy usually requires thin sections of tissue, tissue clearing allows for deep imaging of large volumes of intact tissue. In the brain, this allows for the visualization of complete cell networks in exquisite detail without the need for thin slices (Voleti et al., 2019). Tissue clearing techniques, such as CLARITY (Tomer et al., 2014), CUBIC (Susaki et al., 2015) and iDISCO (Renier et al., 2014) involve removing light-refractive compounds such as lipids while retaining any fluorescent markers for cell visualization. Dr Hillman described her group’s recent development of the HOLiS imaging system (human brain-optimized light sheet) which leverages SCAPE’s single objective light sheet geometry to enable high-speed imaging of > 5 mm thick, cleared samples of unlimited lateral extent. She described progress on a collaboration to use HOLiS to image the entire human brain at cellular resolution.

### Optical Coherence Tomography

Dr. Hui Wang presented work on the application of optical coherence tomography (OCT) and its growing application in brain mapping. OCT uses coherent, backscattered light to analyze tissue types and structure. Standard OCT scans are capable of visualizing tissue with approximately 1–15 µm resolution, making this technology a useful choice for mapping neuroarchitecture and connectivity within the brain. At this resolution, OCT is capable of bridging the gap between macroscale, whole-brain MRI scans and microscale, histological images (Wang et al., 2018). While higher resolution optical imaging such as OCT have limited field of view and thus are low throughput, polarization sensitive imaging allows for µm resolution 3D visualization of fibers which can help overcome reconstruction challenges in locations where tractography techniques struggle. Polarization sensitive OCT (PS-OCT) measures the polarization state of light in addition to the optical density of the tissue, providing data on additional properties of biological tissue including the birefringence and optic axis. Visualizing the optic axis is highly valuable for neuroimaging applications, as the optic axis reveals the directionality of tissue structure. This type of data is a key feature in studying fiber connectivity, white matter tracts, vasculature, and other neural features with high levels of directionality and organization. Earlier dMRI techniques often struggle to differentiate fibers at regions of high connectivity and at locations of fiber crossings, branching, fanning, and turning. This microscale technique is well suited to address some of these challenges.

Dr. Wang has worked on fully automatic serial sectioning PS-OCT (as-PSOCT) to study fiber orientation throughout large sections of the brain in a highly efficient manner (Wang et al., 2018). The development of as-PSOCT imaging enabled automatic, vibratome sectioning of human, ex vivo brain tissue at an imaging resolution of 3.5 µm and a tractography resolution of 30 µm, revealing fiber networks at a greater resolution than dMRI techniques. Dr. Wang has also used a serial optical coherence scanner (SOCS) to distinguish gray and white matter, delineate fiber architecture throughout the brain, and apply a structure tensor (ST) model to ex vivo rat brain images to construct 2D and 3D quantitative fiber orientation maps (Wang et al., 2014). Using SOCS to create volumetric scans of brain tissue fibers illustrates connectivity at a mesoscale level and allows for connection with histology images. Registration with histology is a crucial step to link broader connectivity relationships to the underlying microstructure and provide multiscale assessment of tissues. Dr. Wang’s ongoing research on the use of 3D PS-OCT for quantifying fiber orientation in the brain can be found in a recent paper (Liu et al., 2023). Dr. Nathan Blanke presented an abstract on the use of PS-OCT with birefringence microscopy analyze ex vivo myelinated axons in both the mesoscale and microscale.

## Discussion/Conclusion

The past decade has seen rapid advances in human imaging technology that are now pushing the limits of sensitivity and resolution beyond what first-generation systems offered, rivaling capabilities that were previously relegated to the realm of microscopy and expanding the frontiers of noninvasive imaging to enable deeper mechanistic studies of human biology and disease. The symposium highlighted key technologies that the Center for Mesoscale Mapping, Martinos Center, and Neuroimaging Training Program have been at the forefront of developing. We envision that these next-generation imaging technologies, and the trainees experienced in their application, will allow us to see into the human brain and body with unprecedented spatial and temporal resolution, opening new vistas with respect to the biological processes that can be visualized and measured in living humans. Evidence for current and future clinical translation was especially impactful as there are immense opportunities for developing mesoscale imaging biomarkers for diagnosis of brain-based disorders and tracking treatment response. Mesoscale imaging has and will continue to provide much greater understanding of brain structure and function in health and disease. Many of the most exciting mesoscale specific results concerned the insights gained from optical imaging acquisitions, which allow for simultaneous, direct mapping of both hemodynamics and neural activity, demonstrating unequivocally that BOLD fMRI is indeed capturing salient information about brain activity during both task and ‘﻿rest’ states. Many challenges and obstacles remain, for example, extending acquisition and analysis pipelines for human brain imaging to consistently include the cerebellum. We look forward to the opportunities that emerging mesoscale imaging technologies will unveil, providing new insights into human illness and fundamental questions in biology.

Both trainees and faculty benefitted from this significant educational opportunity. Collaborative planning between faculty and trainees provided the opportunity for trainees to learn how to plan a scientific event. Many NTP trainees who presented posters and gave “flash﻿”﻿ oral presentations especially benefited from the symposium: in addition to gaining experience from presenting their work, they had opportunities throughout the day to engage in one-on-one discussions with visiting scientists and other faculty, potentially opening the door to future collaborations. Finally, the preparation of this this comprehensive, multidisciplinary review manuscript reporting on the scientific frontiers that were presented and discussed during the symposium was an outstanding learning opportunity for trainees. Trainee author contributions were based both on their own research expertise and domains outside of their primary field since they had the unique, providing the opportunity to work one-on-one on content with faculty experts outside of their graduate mentorship team. Active work on the development of the symposium and manuscript provided trainees to with new scientific experiences, enabling and inspiring the next generation of neuroimaging investigators.

## Data Availability

No datasets were generated or analysed during the current study.
